# The Dynamics of Autism Spectrum Disorders: How Neurotoxic Compounds and Neurotransmitters Interact

**DOI:** 10.3390/ijerph10083384

**Published:** 2013-08-06

**Authors:** Ilona Quaak, Madeleine R. Brouns, Margot Van de Bor

**Affiliations:** Health and Life Sciences, Faculty of Earth and Life Sciences, VU University, De Boelelaan 1085 T-634, 1081 HV Amsterdam, The Netherlands; E-Mails: m.r.brouns@vu.nl (M.R.B.); margot.vande.bor@vu.nl (M.V.B.)

**Keywords:** autism spectrum disorder, ASD, neurotoxic exposure, organochlorine, organophosphate, phthalate, neurotransmitter, GABA, glutamate, serotonin, dopamine

## Abstract

In recent years concern has risen about the increasing prevalence of Autism Spectrum Disorders (ASD). Accumulating evidence shows that exposure to neurotoxic compounds is related to ASD. Neurotransmitters might play a key role, as research has indicated a connection between neurotoxic compounds, neurotransmitters and ASD. In the current review a literature overview with respect to neurotoxic exposure and the effects on neurotransmitter systems is presented. The aim was to identify mechanisms and related factors which together might result in ASD. The literature reported in the current review supports the hypothesis that exposure to neurotoxic compounds can lead to alterations in the GABAergic, glutamatergic, serotonergic and dopaminergic system which have been related to ASD in previous work. However, in several studies findings were reported that are not supportive of this hypothesis. Other factors also might be related, possibly altering the mechanisms at work, such as time and length of exposure as well as dose of the compound. Future research should focus on identifying the pathway through which these factors interact with exposure to neurotoxic compounds making use of human studies.

## 1. Introduction

In recent years concern has risen about the increasing prevalence of developmental disorders. A striking example is Autism Spectrum Disorders (ASD). ASD is a generic term for a group of neurodevelopmental disorders primarily diagnosed in childhood. The disorders are characterized by impaired social interaction, communication and repetitive behavior.

In the past decades, the prevalence of ASD has shown rapid growth. From 2007 to 2011–2012, the incidence of ASD rose from 1.16% to 2.00% in the United States of America [1]. This rapid increase is observed not only in the United States of America, but in other parts of the globe as well. In the United Kingdom, the prevalence of ASD has risen from 4.4 per 10,000 between 1966 and 1991 to 12.7 per 10,000 between 1992 and 2001 [2], with a current estimate as high as 157 per 10,000 [3]. An increase in prevalence has also been found in Sweden [4]. Hence, the increasing prevalence of ASD appears to be a worldwide phenomenon.

Although the increase might reflect a genuine rise in the prevalence of ASD, some researchers claim that the rise can be attributed to increased awareness of the disorders, while others assert that changed diagnostic tools and a broadening of the diagnostic criteria are accountable. Nevertheless, an increasing number of authors believe that these explanations are not sufficient to explain the rapid increase of ASD [5].

The increased prevalence demands a closer look at the etiology of ASD, which remains to be elucidated. Evidence implicates that genetic factors are involved. For example, the prevalence rate of ASD under boys is approximately four times higher than under girls [6]. In addition, Hallmayer *et al*. found an ASD concordance of 77% among male monozygotic twins, while concordance was 31% among male dizygotic twins. For females, concordance was 50% in monozygotic twins and 36% in dizygotic twins [7]. Moreover, ASD has regularly been linked to genetic malfunctioning in the past, such as copy number variations [8] and genes encoding molecules that regulate cell-adhesion [6]. In a recent review by Xu *et al*. [9], more than 434 high-confidence genes were related to the development of ASD.

Most authors nowadays agree that both genetic and environmental factors play a role in the development of ASD. For example, high concordance of ASD among boys and girls cannot be explained by genetic heritability alone; shared environmental factors explain a large proportion of the variance in liability [7]. In addition, complex disorders such as ASD present themselves in different ways, *i.e*., a heterogeneous pattern of symptoms can be seen within the same disorder [10]. Moreover, the rapid increase in the prevalence of ASD as seen in the past decades cannot be explained by genetic influences alone. A significant contribution from environmental influences provides a plausible explanation [11]. It is hypothesized that heritable factors might contribute to susceptibility for ASD, creating sensitivity of the developing brain to adverse effects of environmental factors [12].

In recent years, special attention has been given to the influence of exposure to neurotoxic compounds. Neurotoxic compounds are natural or artificial substances that influence the functioning of the nervous system. Many products used on a daily basis contain compounds that are suspected of having properties that affect neurodevelopment [13]. For example, phthalate esters such as di(2-ethylhexyl) phthalate (DEHP), dicyclohexyl phthalate (DCHP) and dibutyl phthalate (DBP) are used as plasticizers in the production of electronics, packaging and children’s toys. Organochlorines such as endosulfan, DDT and dieldrin are used as pesticides, while organophosphates such as chlorpyrifos, diazinon, parathion and dichlorvos are used as insecticides for agricultural purposes. Hence, human exposure to neurotoxic compounds is widespread and moreover, has generally increased from the 1980s to 2000s [10]. As human health might be influenced by neurotoxic compounds, it is crucial to investigate the effects of these compounds.

Over the last decades, accumulating evidence has shown that exposure to neurotoxic compounds is related to deviant neurodevelopment. Exposure to polychlorinated biphenyls (PCBs) can cause neurodevelopmental disorders, as well as subclinical brain dysfunction [13]. More specifically, non-dioxin-like PCBs alter Ca^2+^-dependent signalling pathways related to ASD [10]. Acute exposure to the non-dioxin-like PCB95 stimulates a bursting type of Ca^2+^-activity which has been observed in neurons expressing mutations in genes that have been related to ASD susceptibility [14]. Remarkably, in recent years the use of acetaminophen in children has been related to an increased risk of developing autism. For example, in a parent survey the use of acetaminophen after measles-mumps-rubella vaccination was related to autistic behaviour in children up to 5 years of age [15]. Moreover, exposure to acetaminophen has been found to alter social behaviour in mice [16]. Schultz *et al*. [17] hypothesize that acetaminophen might activate the endocannabinoid system which during development can have neuromodulatory consequences, possibly triggering autism. Indeed, cannabinoid receptor type 2, but not type 1, has found to be upregulated in blood cells of children affected by ASD [18].

In addition, prenatal exposure to organophosphates has been related to a significant reduction in childhood IQ [19,20]. In a cohort study, children who were exposed prenatally to the organophosphate pesticide chlorpyrifos showed a decline in cognitive scores during preschool years [21]. Children exposed to higher levels of chlorpyrifos were more likely to score within the clinical range of the scales Attention Problems, Attention Deficit Hyperactivity Disorder and Pervasive Development Disorders of the Child Behavior Checklist than children exposed to lower levels. In addition, both prenatal and postnatal exposure to organophosphates has been linked to a higher risk of developing a Pervasive Development Disorder [22]. In a review, De Cock *et al*. [23] reported positive associations between ASD and perinatal exposure to several neurotoxic compounds, including organochlorine and organophosphate pesticides and phthalates. In particular, Roberts *et al*. [24] reported that during the gestational period, living near sites where pesticides are being used for agricultural purposes increases the risk of the foetus to develop ASD by six times (odds ratio of 6.1).

As certain neurotoxic compounds can pass placental barriers, exposure can start as early as the prenatal period. During this period the embryo and fetus are continuously subjected to rapid cell division, cell differentiation [25] and organogenesis. Within the first nine months of life, the human brain develops from a strip of cells into a complex organ comprising billions of specialised cells that are highly interconnected and located with precision [13]. Hence, exposure to neurotoxic compounds might be particularly harmful for neurodevelopment during this stage. However, human brain development continues postnatally. Glial cell growth and axon myelination proceed for years and synaptic modelling extends through childhood and adolescence [13,26], prolonging the period of heightened vulnerability to neurotoxic compounds.

The question remains through which mechanisms neurotoxic compounds cause ASD. The field of neurotoxicology regards that neurotoxic compounds disturb neurological functions by interference with the function or structure of neural pathways, systems and circuits [27]. Interference with neurotransmitter functioning might play a key role, as research has shown a connection between, on the one hand, neurotoxic compounds and altered neurotransmitters functioning, and, on the other hand, altered neurotransmitter functioning and the occurrence of ASD. For example, Fatemi *et al*. report that several neurotransmitters, such as γ-aminobutyric acid (GABA), glutamate (Glu) serotonin (5-HT) and dopamine (DA) are related to deficits in autism, a type of ASD [28]. Both levels and associated proteins, such as receptors and transporters of the neurotransmitters are associated with the disorder. In the following section, these individual neurotransmitters will be discussed in light of their relation to ASD.

*GABA*: In a study by Harada *et al*. [29], low levels of GABA were observed in the left frontal lobe of subjects suffering from autism when compared with controls. Anomalies on chromosome 15 have been associated with the development of ASD and deficiencies in GABA_A_ receptors. In addition, genes GABRB1, GABRB3, GABRA4, GABRA5 and GABRG3 encoding GABA_A_ receptors have been associated with ASD with high confidence (as reviewed by [9]). In a case study, Hogart *et al*. [30] found that two subjects diagnosed with ASD showed dysregulation of GABA_A_ receptor subunit genes GABRB3, GABRA5, and GABRG3 located on chromosome 15q11-13. Anomalies in this cluster are likely to negatively influence the maturation of local circuits which are implied in information processing and complex cognitive behavior [31]. Also, in a postmortem study by Blatt [32], GABA_A_ and GABA_B_ receptor expression was significantly decreased in the cerebellum. Fatemi *et al*. [33] reported that subjects suffering from ASD present decreased levels of GABBR1 in the brain and that GABAergic dysfunction is widespread in ASD cerebella. The authors suggest that altered gene expression of genes related to GABA might be related to cognitive impairments associated with the disorder.

*GLUTAMATE*: In a review by Xu *et al*. [9], ASD was related to genes GRIK2, GRIN3B and GRIA3, encoding Glu receptors. In addition, anomalies in regions on chromosomes six and seven, encoding Glu receptors, have been related to ASD [34,35]. GAD1, encoding glutamic acid decarboxylase which converts Glu into GABA, has also been associated with ASD. Moreover, decreased levels of glutamic acid decarboxylase have been observed in subjects diagnosed with autism [36,37] although this was not confirmed by a study of Shinohe [38]. Finally, increased Glu levels have been reported in blood plasma and serum of subjects with ASD [38,39]. Abnormalities in the glutamatergic system might therefore be implied in ASD. Indeed, epileptic seizures which have been related to excitatory Glu and decreased GABA, are common in ASD [40].

*SEROTONIN*: The association between 5-HT and ASD is intriguing. Multiple, often rare alleles at the 5-HT transporter locus have been related to an increased risk of developing ASD [41]. Genes SL6a4 and ASMT encoding a 5-HT transporter and the enzyme acetylserotonin O-methyltransferase have also been related to the disorder (as reviewed by [9]). Hyperserotonemia, increased 5-HT levels, is a phenomenon commonly observed among subjects with ASD, reported in about 25% of the subjects [42]. However, selective serotonin reuptake inhibitors (SSRIs) such as fluoxetine, fluvoxamine and venlaxafine are prescribed in subjects with a form of ASD and show positive effects on stereotyped and repetitive behavior, interests, social deficits and communication problems [43]. Nevertheless, SSRIs seem to be less efficacious and are more poorly tolerated by children suffering from ASD than by adults with the same diagnosis [44]. In addition, positive effects of SSRIs on social relatedness have not been found in placebo-controlled studies [45]. Moreover, in recent studies the use of SSRIs by pregnant women has also been associated with an elevated risk of developing ASD in their children [46]. Although confounding by indication might play a role, it could be hypothesized that prenatal exposure to SSRIs leads to disturbed 5-HT levels later in life. These findings have led to an increased interest in the role of the serotonergic system in the etiology of ASD [47].

*DOPAMINE*: There is evidence that anomalies in the dopaminergic system are related to deficits as observed in subjects diagnosed with ASD [48]. Increased dopamine transporter (DAT) levels have been found in the orbitofrontal cortex in subjects with high functioning autism, using positron emission tomography (PET) [49]. Subjects with ASD also present elevated levels of homovanillic acid in urine, a dopamine metabolite [50], which might be indicative of increased DA turnover. In addition, genes DRD3, DRD4 and DBH have been related to ASD, encoding DA receptors and the enzyme dopamine beta-hydroxylase, which converts DA to norepinephrine.

The dopaminergic system has been related to executive functioning, such as analyzing, planning and prioritizing [51]. Children with ASD show deficits in this area compared to children developing normally, as illustrated by a study of Happé *et al*. [52]. A group of children with and without ASD was matched by age and IQ and subsequently compared on a battery of tasks associated with executive functioning, including response/selection, planning/working memory and flexibility. Children with ASD scored lower on most of the tasks, even though a significant difference was only found on the scale response/selection.

In addition, the dopaminergic system has been related to motor activity, social behavior, attentional skills and perception, while abnormal development in these three areas have all been associated with ASD as well [51,53]. Medications prescribed to subjects with ASD include haloperidol and risperidone, which are antipsychotic medications acting as DA antagonists. In a study by McCracken and colleagues [48], treatment with risperidone was associated with improvements in stereotypic behavior, irritability and hyperactivity. In addition, some SSRIs targeting both 5-HT and DA receptors might have clinical benefits [54]. Hence, although there are no indications that DA levels are increased respectively decreased in subjects diagnosed with ASD, administration of DA antagonists may have beneficial effects.

As presented above, GABA, Glu, 5-HT and DA seem to be implicated in the pathology of ASD. In addition, ample literature is available on the effects of neurotoxic compounds on the functioning of neurotransmitters. Organophosphates are well-known to inhibit the enzyme acetylcholinesterase, which results in accumulation of acetylcholine in the synapses and an excessive stimulation of cholinergic neurons [55]. Disruption in acetylcholine neurotransmission after exposure to organophosphates might lead to alterations of other neurotransmitter systems as well [56]. In addition, exposure to organochlorine pesticides has been related to inhibition of GABA_A_ receptors [57]. The organochlorine endosulfan prevents binding of GABA to its receptor site, leading to uncontrolled neuron excitation [58]. Exposure to PCBs has been related to both increased and decreased DA levels and inhibits DAT [59,60,61,62]. Also, Yang *et al*. [63] found that exposure to the phthalate DEHP modulates the function of GABA_A_ receptors. Moreover, in utero and neonatal exposure to low levels of DEHP might result in changes in dopaminergic nuclei in mice [64].

In the literature, exposure to organochlorines, organophosphates and phthalates has been related to the development of ASD [23]. In addition, the neurotransmitters GABA, Glu, DA and 5-HT have been related to neurotoxic exposure and ASD. Hence, the effect of neurotoxic compounds on the functioning of neurotransmitters might play a key role in the development of ASD and will be the focus of this review. However, although much has been published about the relationship between neurotoxic compounds and neurotransmitters associated with ASD, it is not clear through which mechanisms neurotoxic compounds influence neurotransmitters. As an important step towards unravelling this interaction, we will provide an overview of the literature available about exposure to the neurotoxic classes organochlorines, organophosphates and phthalates and the neurotransmitters GABA, Glu, DA and 5-HT, related to ASD. The aim is to find possible mechanisms and related factors, explaining how exposure to neurotoxic compounds can lead to ASD via neurotransmitter anomalies.

## 2. Experimental Section

A literature search was performed using specific keywords in PubMed. Two types of keywords were combined to find publications, respectively terms related to exposure to neurotoxic substances and terms referring to the selected neurotransmitters. To describe exposure to neurotoxic compounds, the following keywords were introduced in PubMed: organochlorine(s), organochlorine pesticide(s), organophosphate(s), organophosphate pesticide(s), organophosphate insecticide(s), phthalate(s). With respect to neurotransmitters, the following keywords were introduced: GABA, γ-aminobutyric acid, glutamate, dopamine, serotonin. Publications written in English from the last ten years were included (from April 2003 to April 2013) in which a quantification method was used to value the results. A total of 410 publications was retrieved. Despite a careful and thorough literature search and selection, it is possible that not all related literature has been included in the overview. Selection took place by investigating titles, abstracts and the publications themselves. Only outcomes and results relevant to the scope of the current review were included. Direct effects on the selected neurotransmitters have been included in the review, such as effects on neurotransmitter levels, uptake, release and gene expression of receptors and transporters involved. Seven publications were excluded because values of significance were not clear. After selection, a number of 62 publications was included in the current review.

## 3. Results and Discussion

### 3.1. Results

Table 1 presents all neurotoxic compounds that have been included in the current review. Tables S1–S3 present a literature overview of the effects of exposure to respectively organochlorines, organophosphates and phthalates on GABA, Glu, DA and 5-HT.

**Table 1:** Neurotoxic compounds, classified.

Class	Compound
Organochlorines	Dichlorodiphenyldichloroethane (DDD)
	Dichlorodiphenyldichloroethylene (DDE)
	Dichlorodiphenyltrichloroethane (DDT)
	Dicofol
	Dieldrin
	Endosulfan
	Endosulfan sulfate
	Lindane
	Methoxychlor
	Polychlorinated Biphenyls (PCBs; congeners): PCB25, PCB52, PCB77, PCB91, PCB95, PCB101, PCB103, PCB126, PCB138, PCB180, PtCB, TCB, ortho-TCB
	Polychlorinated Biphenyls (PCBs; mixtures): Aroclor (A)1016, A1221, A1242, A1248, A1254, A1260
	Trichloroethylene (TCE)
Organophosphates	Chlorpyrifos
	Diazinon
	Dichlorvos
	Methamidophos
	Methyl parathion
	Monocrotophos
	Paraoxon
Phthalates	Dibutyl phthalate (DCHP)
	Di(2-ethylhexyl) phthalate (DEHP)
	Dibutyl phthalate (DBP)

Complete information is presented with respect to exposure assessment, analysis and outcome measurements. Experimental groups were exposed to most neurotoxic compounds by means of microdialysis, (culture) medium or dissolution in oily food. Within each table, literature is presented in the following order: GABA, Glu, DA, 5-HT. If several neurotransmitter systems are investigated within the same publication, the source is listed in the overview of the neurotransmitter that is most prominent in the publication or, if all are equal, that is first in line in the current overview.

#### 3.1.1. Organochlorines

Table S1 presents an overview of the effects of exposure to organochlorines on GABA, Glu, DA and 5-HT. Although various methods have been used, some general findings can be reported regarding the effect of exposure to organochlorines on neurotransmitter systems. Most of the studies reported elevated GABA levels after exposure to organochlorines, such as PCB52, dieldrin and methoxychlor. However, Cabaleiro *et al*. [65] reported increased GABA levels in the prefrontal cortex of male Sprague-Dawley rats at postnatal day 30 (PND30) and decreased GABA levels at PND60 after exposure to 6.12 mg/kg/day of endosulfan. With respect to gene expression, Dickerson *et al*. [66] reported an upregulation of gene expression of GABA_B_ receptor 1 in female Sprague-Dawley rats after exposure to 1 mg/kg of Aroclor1221 or 1 mg/kg of a PCB mixture (PCB138, PCB153, PCB180). This finding was not observed in male rats. Male Sprague-Dawley rats presented a significant downregulation of gene expression of GABA_B_ receptor 1 after exposure to the PCB mixture, while the expression of the GABA_B_ receptor 2 was upregulated after exposure to the same compound.

With respect to Glu, exposure to 25 mg/kg of methoxychlor for 30 days increased striatal Glu levels in adult male Sprague-Dawley rats [67]. The findings of Cabaleiro *et al*. regarding exposure to endosulfan and GABA levels were similar to those reported with respect to Glu levels in the prefrontal cortex. After an exposure of 6.12 mg/kg/day of endosulfan Glu levels increased at PND15 and PND30, but no effect was found at PND60 [65]. Exposure to Aroclor1254 was related to decreased hippocampal Glu levels and Glu uptake in astroglial fractions of glial plasmalemmal vesicles, while both an increase and decrease in synaptosomal Glu uptake were reported [68,69,70]. Also, after exposure to 1 mg/kg/day of PCB52 from gestational day 7 (GD7) to PND21, Boix *et al*. reported increased Glu levels in male Wistar rats, while the same author has reported decreased Glu levels in a more recent study using the same treatment group, dose and length of exposure [71,72]. However, different techniques were used for the analysis of the data, respectively microdialysis and High-Performance Liquid Chromatography (HPLC). Finally, exposure of Sprague-Dawley rats to 1 mg/kg of a mixture of PCB138, PCB153 and PCB180 was related to downregulation of Glu receptors gria2 and grin2a in male rats, while Glu receptor grin2c was upregulated in female rats [66].

A total of 20 studies was selected with respect to the effects of organochlorines on the DA system, including level, uptake, release, turnover and gene expression. In an *in vitro* study by Dreiem *et al*. [61], Long-Evans rat striatal synaptosomes were exposed to a dose of 10, 20 or 40 μM of a mixture of Aroclors 1242, 1248, 1254 and 1260 on PND7, 14 or 21. Exposure to the mixture reduced DA level in the synapses, although elevated extraneuronal (medium) DA levels were reported. These findings were consistent with an *in vitro* study by Lyng *et al*. [62], in which co-cultures of the ventral mesencephalon, striatum and tissue culture medium of fetal Sprague-Dawley rats were exposed to the same mixture of Aroclors, with a dose of 2 μM or 8 μM for 1, 3, 7 or 14 days. Again, exposure to the mixture was related to increased extraneuronal (medium) DA levels, while decreased DA levels in co-cultures of the striatum and ventral mesencephalon were reported, which were accompanied by a reduction of DA neurons and DAT proteins. In addition, Caudle *et al*. [73] reported reduced DAT levels and DA binding after exposure of adult C57BL/6J mice to a mixture of 7.5 or 15 mg/kg/day of Aroclors 1254 and 1260 for 3,7,14 or 30 days. These effects were reported after 14 and 30 days of exposure.

With respect to PCBs, delayed effects on DA were observed after exposure as well. Honma *et al*. [74] reported an initial increase of DA levels in the whole brain of one week old Sprague-Dawley rats after exposure to 16 mg/kg/day of PCB153 from GD10 to GD16, which was followed by a decrease in DA turnover in rats that were nine weeks of age. Although the results were not significant, the authors report that the initial increase of DA levels was followed by a decrease in DA concentration, metabolism and turnover, which were associated with a lower activity of DA neurons. This finding was not consistent with a study by Boix *et al*. [72], who reported elevated extracellular DA levels in Wistar rats of four months old, after exposure to 1 mg/kg/day of PCB180 from GD7 to PND21.

With regard to 5-HT, Lafuente *et al*. [75] reported that a dose of methoxychlor of 25 mg/kg/day for one month was related to increased 5-HT levels in the anterior hypothalamus (*p* ≤ 0.05) and a decrease in 5-HT turnover in male Sprague-Dawley. However, exposure to methoxychlor reduced 5-HT levels in the posterior hypothalamus (*p* ≤ 0.05). No effects on 5-HT were found in a second study by the same authors, investigating effects of methoxychlor exposure in the striatum, using the same population, dose, length of exposure and analysis techniques [67].

No effects on gene expression of 5-HT receptor HT1A were reported after exposure to a mixture of PCBs 28, 52, 101, 138, 153 and 180 [76]. In a study by Honma *et al*. [74], Sprague-Dawley rats were exposed to 16 or 64 mg/kg of PCB153 from GD10 to GD16. As rat brain size was too small at postnatal week one to separate brain regions, whole brain 5-HT levels were observed at this time point. The authors reported an increase in whole brain 5-HT levels after exposure to 64 mg/kg/day in one week old rats, followed by a decrease in 5-HT turnover in the hindbrain when rats were nine weeks old. These findings are similar to results from a study in male Sprague-Dawley rats in which 5-HT levels were elevated at PND30 and 60 after exposure to 6.12 mg/kg/day of endosulfan from GD7 until PND21. The increase of 5-HT levels at PND60 was accompanied by decreased 5-HT turnover [65].

#### 3.1.2. Organophosphates

Table S2 presents an overview of the effects of exposure to organophosphates on GABA, Glu, DA and 5-HT.

Out of four studies that were included on the effects of exposure to paraoxon on GABA uptake, three reported a significant reduction. Pourabdolhossein *et al*. [77] reported a significant decrease in GABA uptake after exposure of male Wistar rat synaptosomes to 0.1 or 1 μM of paraoxon for ten minutes. These results were confirmed by a study by Mohammadi *et al*. [78], reporting a significant decrease of GABA uptake by synaptosomes derived from the hippocampus and cortex of adult male Wistar rats after exposure to 0.1, 0.3 and 0.7 mg/kg of paraoxon. However, an increase in synaptosomal GABA uptake was reported after exposure of synaptosomes from male Wistar rats to lower concentrations (10^−8^–10^−6^ M) of paraoxon for 20 min while GABA uptake was decreased after exposure to higher concentrations (10^−5^–10^−3^ M).

In a study by Noriega-Ortega *et al*. [79], 2.6 mg/kg of methamidophos was administered to male BALB/c mice every three days, until the mice were three, six or nine months old. Exposure to methamidophos was related to a decrease of GABA release in the cerebral cortex after three months, while an increase of GABA release in the same brain area was observed after nine months. The authors associated the decrease of GABA release with convulsive seizures that mice presented after neurotoxic exposure.

With regard to Glu, effects of chlorpyrifos exposure on Glu levels were investigated in two studies. In a study by Montes de Oca *et al*. [80] adult male Lister Hooded rats were exposed to a single dose of 250 mg/kg of chlorpyrifos. Analyses were executed 15 months after exposure, showing decreased Glu levels in the striatum. Contrarily, Rush *et al*. [81] reported elevated extracellular Glu levels in cortical cell cultures from Swiss Webster mice after exposure to 100 μM of chlorpyrifos for six hours. However, besides a different dose and length of exposure, different techniques were used for the analysis.

Nine studies investigating the effects of chlorpyrifos on the dopaminergic system have been included in the current review. None of the studies reported significantly elevated neurotransmitter levels after exposure to the neurotoxicant, despite the use of various doses and techniques for analysis, and the different moments and lengths of exposure to the neurotoxicant used in the various studies. After chlorpyrifos exposure, decreased DA levels were reported in the hippocampus, cerebral cortex and striatum in animal studies [82,83,84,85,86,87]. These findings were consistent with results from a study investigating cell cultures of PC12 cells [88]. In several studies, a decrease in DA levels was accompanied by an increase in DA turnover [82,84,86].

The reported decreased DA levels after chlorpyrifos exposure are consistent with studies on dichlorvos. Binukumar *et al*. [89] exposed male Wistar rats to 2.5 mg/kg/day of dichlorvos for 12 weeks. Analyses of DA levels were carried out in the substantia nigra and corpus striatum using HPLC. A significant decrease in DA levels in the substantia nigra and corpus striatum was reported. A more recent study by the same authors, also using male Wistar rats and the same dose, length of exposure and analysis techniques, has shown similar results [90].

Regarding 5-HT, two studies reported decreased neurotransmitter levels after chlorpyrifos. In a study by Eddins *et al*. [84], zebrafish were exposed to 0.29 μM of chlorpyrifos from two hours to five days postfertilization. A significant decrease of 5-HT levels was found in six day old zebrafish, using HPLC. In addition, Slotkin *et al*. [91] reported reduced 5-HT levels after exposure to 5 mg/kg/day of chlorpyrifos administered from PND11 to 14, in Sprague-Dawley rats. The decrease in 5-HT levels on PND21 in the brainstem was accompanied by an increase in 5-HT turnover. In contrast, the same authors [92] report a main exposure effect (increasing) of chlorpyrifos on 5-HT levels in the striatum in Sprague-Dawley rats who had been exposed to 1 or 5 mg/kg/day of chlorpyrifos during GD9 to GD12, or GD17 to GD20.

 In a different study, sea urchins were exposed to 0.01, 0.10 or 1.00 mg/L of monocrotophos during the first 12, 15, 18, 24 or 30 h postfertilization [93]. An initial increase in gene expression of the 5-HT transporter was observed at the 24 and 30 h postfertilization stages, followed by a decrease at the 36 and 48 h postfertilization stages.

#### 3.1.3. Phthalates

In Table S3, an overview is presented of the effects of exposure to phthalates on GABA, Glu, DA and 5-HT. In several studies, authors made use of ratios to quantify the results. Results with a ratio higher than 1.50 or lower than 0.50 (*i.e*., more than 50% change) are reported in Table S2. With regard to neurotransmitter levels, only literature investigating GABA levels was available. In a study by Carbone *et al*. [94], Wistar rats were exposed to 3 or 30 mg/kg/day of DEHP, from GD1 to weaning. Animals were sacrificed at PND15, when analyses were carried out using HPLC. A significant decrease in GABA levels was found in male rats after exposure to 30 mg/kg/day of DEHP, while female rats presented an increase in GABA levels. In a second study by Carbone [95], the same methods were used, only this time, rats were exposed for 30 days: an increase in GABA levels in male rats was reported.

The remaining studies focused on the effects of phthalates on gene expression of proteins related to the selected neurotransmitters. The findings of these studies are diverse, showing upregulation and downregulation of related receptors.

### 3.2. Discussion

The objective of this review was to investigate the evidence for the hypothesis that organochlorines, organophosphates and phthalates influence the functioning of GABA, Glu, DA and 5-HT, possibly causing ASD. Hence, literature on the effects of exposure to the selected neurotoxic compounds on the neurotransmitters has been presented. In this section we will interpret these findings and propose possible mechanisms of action and related factors.

#### 3.2.1. GABA

In literature, ASD has been associated with decreased GABA levels [29]. In the current review reduced GABA levels were reported after exposure of male Sprague-Dawley rats to 0.61 or 6.12 mg/kg/day of the organochlorine endosulfan during the same period (GD1-PND21). The reduction in GABA levels was observed with a delay, as decreased GABA levels were reported at PND60, 39 days after endosulfan exposure had ended. In addition, decreased GABA levels were observed after exposure of adult male Lister hooded rats to a single dose of 250 mg/kg of the organophosphate chlorpyrifos Furthermore, in a study by Noriega-Ortega *et al*. [79] male BALB/c mice were exposed to 2.6 mg/kg of methamidophos every three days resulting in a decrease of GABA release in the cortex and hippocampus after three and six months. An exposure to 30 mg/kg/day of the phthalate DEHP throughout gestation until weaning (GD1-PND21) also resulted in decreased GABA levels in prepubertal male rats.

ASD has been related to a dysregulation of both GABA_A_ and GABA_B_ receptor expression. Blatt *et al*. [32] reported a significant decrease of GABA_A_ and GABA_B_ receptor expression in the cerebellum. In addition, mutations of genes encoding GABA receptors have been related to ASD with high confidence (as reviewed by [9]).

In the current review, significant downregulation of GABA_B_ receptor 1 (GABBR1) gene expression was reported after exposure of Sprague-Dawley rats during GD16 and GD18 to 1 mg/kg of a PCB mixture, including PCB138, PCB153 and PCB180 [66]. Male Wistar rats exposed to 29 μg of the phthalate DCHP on PND5 presented dysregulation of both GABA_A_ and GABA_B_ receptors and transporters gene expression [96]. In a different study, exposure to the phthalate DEHP resulted in downregulation of GABA transported Gat3.

Results from the current review have shown that exposure to neurotoxic compounds can result in changes in the GABAergic system that have been related to ASD. Exposure to neurotoxic compounds may directly influence the GABAergic system. However, it could also be hypothesized that exposure to neurotoxic compounds leads to altered expression of genes that have been related to both ASD and the GABAergic system, for example 15q11-13. Indeed, Tanaka *et al*. [97] propose that the GABRB3 subunit on chromosome 15q11.2-q12, which has been related to ASD, is regulated by epigenetic modulations. Similarly, Hogart *et al*. [30], propose that downregulation of the GABRB3 receptor genes might result from epigenetic alterations.

#### 3.2.2. Glutamate

ASD has been related to genes which encode Glu receptors and the enzyme glutamate decarboxylase (as reviewed by [9]). In the current review, adult male Wistar rats exposed to 10 mg/kg of the PCB mixture Aroclor1254 for 14 days presented alterations in gene expression of Glu transporters EAAC1 and GLT-1 in the forebrain and cerebellum [70]. After exposure to 100 μM of sarin, significant upregulation of Glu receptor GRIA1 was reported in the adult male Sprague-Dawley rat brain [98]. In addition, exposure of male Wistar rats to single doses of the phthalates DCHP and DEHP resulted in gene expression alteration of 16 Glu receptors and transporters, including GRIA3, which in previous research has specifically been related to ASD (as reviewed by [9]).

In a study by Shinohe *et al*. [38], increased Glu levels were observed in adult subjects diagnosed with ASD. In the current review, elevated Glu levels in the cerebellum were reported in male Wistar rats after exposure to PCB52 from GD7 to PND21, although not supported by findings in female Wistar rats. In addition, all studies in which exposure to the organochlorine endosulfan was under investigation, presented an increase in Glu levels. Earth-worms exposed to 0.1, 1.0 or 10.0 mg/kg of endosulfan for seven days showed an increase in Glu levels four days after exposure ended. Similar findings were observed after exposure to 0.1 or 10.0 mg/kg of endosulfan sulfate.

In a study by Cabeleiro *et al*. [65] prefrontal Glu levels in male Sprague-Dawley rats were increased at PND30 after exposure to 0.61 or 6.12 mg/kg of endosulfan throughout gestation until weaning (GD1-PND21). The increase in Glu levels was accompanied by a decrease in GABA levels. In addition, increased striatal Glu levels were found in a study in which adult male Sprague-Dawley rats were exposed to 25 mg/kg of the organochlorine methoxychlor for 1 month. Finally, six hours of exposure of cortical cell cultures from Swiss Webster mice to 100 μM of the organophosphate chlorpyrifos resulted in increased extracellular Glu levels in pure and mixed neuronal and glial cultures.

The anomalies observed in the glutamatergic system of subjects with ASD might be related to deficits in the GABAergic system. Glu is converted to GABA by the enzyme glutamate decarboxylase. Serum levels of Glu were found to be increased in adult subjects with an ASD [38,39], while GABA levels were found to be decreased [29]. Harada *et al*. [29] hypothesize that deterioration in quality of sensory information as observed in subjects with ASD might be caused by an excess in glutamatergic activity combined with the inhibitory influence of GABA. A dysregulation of the GABA/Glu ratio might result in an excess in glutamatergic hyperfunction, altering growth and connectivity of neurons at critical time points. Indeed, Harada *et al*. [29] demonstrated that subjects with an ASD present a decreased GABA/Glu ratio. It is therefore speculated that exposure to neurotoxic compounds might influence the GABAergic/glutamatergic system, resulting in deteriorated information processing as observed in subjects diagnosed with ASD.

#### 3.2.3. Serotonin

Hyperserotonemia or an increase in 5-HT levels, is a phenomenon commonly observed among subjects with ASD, reported in about 25% of the subjects [42]. In this review, increased 5-HT levels were observed after exposure to endosulfan, PCB153, methoxychlor, monocrotophos and chlorpyrifos. In a study by Cabaleiro *et al*. [65], increased 5-HT levels were reported on PND30 and PND60, after exposure of male Sprague-Dawley rats to 0.61 mg/kg and 6.12 mg/kg of the organochlorine endosulfan throughout gestation until weaning (GD1-PND21). The increase in 5-HT levels was accompanied by a reduction in 5-HT turnover. In addition, Sprague-Dawley rats exposed to 16 or 64 mg/kg of PCB153 from GD10 to GD16 presented increased 5-HT levels when they were one week old. Again, the increase in 5-HT levels was accompanied by a decrease in 5-HT turnover.

With regard to organophosphates, elevated 5-HT levels were observed in adult male Sprague-Dawley rats after exposure to 25 mg/kg of methoxychlor for one month, again accompanied by a reduction in 5-HT turnover. Slotkin [92] reported a significant positive main treatment effect of chlorpyrifos exposure on 5-HT levels in the striatum on PND30 after Sprague-Dawley rats had been exposed to the organophosphate from GD17 to GD20. In a second study by Slotkin *et al*. [99], Sprague-Dawley rats were exposed to 0.5 or 2 mg/kg of the organophosphate diazinon from PND1 to PND4. Female rats showed an increase of 5-HT_1A_ transporter binding in female rats, although this finding was not consistent with results in male rats. In a study by Xu *et al*. [93], exposure of sea urchins to 0.01, 0.10 or 1.00 of monocrotophos for 12–48 h postfertilization also resulted in increased 5-HT levels. In the same study, exposure to monocrotophos was also related to an increase in gene expression of the 5-HT transporter. 

In literature, genes SL6a4 and ASMT encoding a 5-HT transporter and enzyme have been related to ASD with high confidence (as reviewed by [9]). In addition, multiple rare alleles related to 5-HT have been associated with ASD [41]. These findings were consistent with results reported in a study by Damodaran [98], who demonstrated an upregulation of SL6a4 after exposure to 50 μg/kg of the organophosphate sarin. The 5-HT transporter Htr6 was also upregulated after exposure to the compound. In addition, upregulation of 5-HT receptors was observed after exposure to 29 μM of the phthalate DCHP.

Findings in the current review have shown that exposure to neurotoxic compounds can result in alterations in the serotonergic system that have been associated with ASD. Therefore exposure to neurotoxic compounds may lead to these alterations such as hyperserotonemia and changed expression of genes related to 5-HT.

#### 3.2.4. Dopamine

Increased DAT levels have been found in the orbitofrontal cortex in subjects with high functioning autism, using PET [49]. Subjects with ASD also present elevated levels of homovanillic acid, a DA metabolite in urine [50], which might be indicative of increased DA turnover.

Increased striatal DAT levels were reported after exposure to 0.3, 1 or 3 mg/kg of the organochlorine dieldrin every three days, for two weeks [100]. The authors also observed an upregulation of DAT gene expression and an increase in DA turnover. In addition, in a study by Padilla *et al*. [101], male Long-Evans rats were exposed to 0, 1 or 5 mg/kg/day of the organophosphate chlorpyrifos for 6 or 12 months. Additional spike doses were administered at 2, 4, 6, 8, 10 or 12 months. After 6 months of chlorpyrifos exposure and additional spikes, an increase in DAT density was observed.

With respect to the studies included in this review, an increase in DA turnover was generally accompanied by a decrease in DA levels. In a study by Lee *et al*. [102], MN9D cells exposed to 20 ppm of Aroclor1254 for 24 h, presented both an increase in DA turnover and a decrease in DA levels. These findings were consistent with a second study by Richardson *et al*. [103], in which C57BL/6J mice were exposed to a single dose of 500 mg/kg of Aroclor1016 or Aroclor1260. Decreased DA levels reported 7 and 14 days after exposure were accompanied by increased DA turnover. However, increased DAT levels were not reported in this study.

In the current review, exposure to organophosphates was related to both decreased DA levels as well as increased DA turnover. In one study, zebrafish were exposed to 0.29 μM of chlorpyrifos from two hours postfertilization to five days postfertilization. A decrease in DA levels was accompanied by an increase in DA turnover in six day old zebrafish, although these findings were not consistent with findings in adult zebrafish. In a study by Moreno *et al*. [86] an increase in striatal DA turnover was reported two days after exposure to 250 mg/kg of chlorpyrifos, followed by a decrease in DA levels in the nucleus accumbens 30 days after exposure.

Genes DRD3, DRD4 and DBH have been related to ASD, encoding DA receptors and a related enzyme (as reviewed by [9]). In the current review, adult male Sprague-Dawley rats exposed to 50 μg of the organophosphate sarin showed significant upregulation of Drd4 gene expression [98]. These findings were consistent with a study in which male Wistar rats were exposed to the phthalate DEHP. After exposure, Drd4 gene expression was upregulated with a ratio of 1.83 compared with controls.

Decreased DA levels as reported in this review might be related to the changes observed in DA receptors. Gerfen *et al*. [104] reported that neurodevelopmental depletion of DA elevates the expression of supersensitive DA receptors. After depletion of DA, the expression levels of DA receptors remain increased. Eddins *et al*. suggest that reduced DA levels in adult fish brains might be implied in long-term behavioral deficits [84]. In addition, treatment with risperidone, blocking DA receptors, has been associated with improvements in stereotypic behavior, irritability and hyperactivity in subjects diagnosed with ASD [48]. Hence, alterations in the dopaminergic system seem to be related to the development of ASD and moreover, findings in the current review indicate that exposure to neurotoxic compounds might result in these alterations.

Summarizing, the findings in the current review support the hypothesis that exposure to neurotoxic compounds can lead to alterations of the GABAergic, glutamatergic, serotonergic and dopaminergic system which have been related to ASD in previous work. However, in several studies, other results were reported that are not supportive of this hypothesis. Hence, other factors might be related, possibly altering the mechanisms at work, which will be discussed below.

#### 3.2.5. Related Factors: Length of Exposure

In a study by Hatcher *et al*. [105] a group of eight weeks old male C57BL/6J mice was given 3 mg/kg of dieldrin every three days for 30 days. HPLC analyses of DAT levels were carried out three days after the end of exposure. The author reported a significant decrease of the DAT level in the striatum. In a similar study of Richardson *et al*. [100], a dose of 3 mg/kg of dieldrin was administered to C57BL/6J mice every three days for two weeks. On PND22, HPLC analyses were carried out, now resulting in a significant increase of the DAT level in the striatum.

The described examples illustrate the importance of the length of exposure. In general, adverse effects after neurotoxic exposure can be divided into two categories: acute or chronic effects [106]. In humans, acute neurotoxicity of organophosphates has been related to adverse effects on the central nervous system and respiratory, cardiovascular, gastrointestinal, motor and sensory problems [107,108]. In contrast, chronic exposure to organophosphates has been related to neurobehavioral changes including anxiety and deficits in learning and information processing as associated with ASD. As the subject grows older, these symptoms can become more pronounced. Neurotoxic exposure continuing for years or even decades might result in neurodegeneration. Long-term exposure to the organochlorines lindane and dieldrin has been related to Parkinson’s Disease [57] and exposure to organochlorine and organophosphate pesticides has been related to an increased risk of Alzheimer’s Disease [109]. Hence, the length of exposure to a neurotoxic compound seems crucial when predicting the final outcome.

#### 3.2.6. Related Factors: Time of Exposure

Another factor possibly of influence is time of exposure. As certain neurotoxic compounds can pass placental barriers, exposure to neurotoxic compounds can start as early as the prenatal period. Some authors claim that during the prenatal period, the fetus is particularly vulnerable because of its rapid growth and development. Moreover, infants are exposed to higher doses of certain neurotoxic compounds [110]. For example, PCBs are accumulated in maternal tissue and are subsequently transmitted to the child by means of breast milk, which results in infant exposure exceeding the exposure of the mother by 100-fold with respect to body weight. High early life exposure might put children at a greater risk of chronic neurotoxic effects than exposure later in life.

In the current review, the relevance of time of exposure can be demonstrated by the study by Hatcher *et al*. [105], as previously mentioned, in which male C57BL/6J mice were exposed to 3 mg/kg of dieldrin every three days for 30 days, resulting in decreased striatal DAT levels. The results were not consistent with a study by Richardson *et al*. [100], which reported an increase in striatal DAT levels at PND22. The mice used in the study by Hatcher *et al*. were young adult male mice (eight weeks old), while mice in the study of Richardson *et al*. were exposed from GD1 to PND21. In a study by Seegal *et al*. [111] mice also showed elevated DA levels after exposure to the PCBs 3,4,3′,4′-tetrachlorobiphenyl (TCB) and 3,4,5,3′,4′-pentachlorobiphenyl (PtCB) from GD1 to PND21, while the same authors state that in a previous study with adult mice no effects had been found. Indeed, central DA function in adult mice might be insensitive to TCB and PtCB while the increases in DA levels found in mice after developmental exposure might reflect a greater sensitivity of the central nervous system in development to certain “estrogenic” PCBs [111]. Hence, the former studies emphasize the importance of time of exposure and critical windows in development.

#### 3.2.7. Related Factors: Dose

With regard to exposure to neurotoxicants, the dose of the compound appears to be of importance. For example, in a study by Ghasemi *et al*. [112] male Wistar rats were exposed for 20 min to either nanomolar (10^−9^–10^−7^) or micromolar (10^−6^–10^−3^) doses of paraoxon. After exposure to nanomolar doses of paraoxon the authors reported significantly increased GABA uptake. In contrast, after exposure to micromolar doses of paraoxon, a significant reduction of GABA uptake was reported.

Exposure to neurotoxic compounds can result in various adverse effects. Some effects are clinically evident, such as visible functional abnormalities which can be presented after high-dose exposure. However, neurotoxic compounds might also exert effects which only can be detected after standard examination. These effects are called subclinical. Therefore a dose-dependent continuum of neurotoxic effects, with clinical and subclinical effects, is proposed [13]. However, recent research has shown that exposure to a higher dose of a neurotoxic compound does not necessarily result in more adverse effects than exposure to a lower dose of the compound. In fact, Vandenberg *et al*. [113] state that exposure to low doses can alter certain endpoints more effectively than higher doses. For example, certain receptors are downregulated after a higher dose, while upregulated at a lower dose [114,115]. Hence, the outcome of exposure to neurotoxic compounds might be related to the dose of the compound under investigation.

## 4. Conclusions

In the current review an overview has been given with respect to neurotoxic exposure and the effects on neurotransmitter systems. The aim was to identify mechanisms and related factors which together might result in ASD. The literature reported in the current review supports the hypothesis that exposure to neurotoxic compounds can lead to alterations of the GABAergic, glutamatergic, serotonergic and dopaminergic system which have been related to ASD in previous work. However, in several studies results were reported that are not supportive of this hypothesis. Hence, other factors might be related, possibly altering the mechanisms at work. Time and length of exposure as well as the dose of the compound seem to be of influence.

With regard to future research it seems crucial to identify the pathway through which these factors interact with exposure to neurotoxic compounds. In addition, the effects of other compounds suspected of affecting neurodevelopment should be investigated, such as acetaminophen. Moreover, future research should investigate the effects of neurotoxic compounds on other neurotransmitter systems possibly related to ASD as well, for example the endocannabinoid system. More human studies are necessary in order to translate findings in animals to humans. Hence, more use should be made of bodily samples from humans, collected both prenatally and postnatally to determine exposure to neurotoxic compounds. A prospective mother-child cohort, following subjects prenatally until adulthood could be used to relate data from neurotoxic exposure to information on behavior development throughout life, focusing on behaviour indicative of ASD. In addition, human risk assessment of developmental neurotoxicity should be improved, using collected exposure data in addition to information from toxicological testing. The significance of differences in dose, time and length of exposure as well as critical windows should be determined with respect to exposure to neurotoxic compounds, especially during early development, focusing on mechanisms to elucidate possible outcomes. In this way, the relation between exposure to neurotoxicants early in life and the onset of ASD in childhood can be further unravelled.

## Abbreviations

5-HT: Serotonin
ASD: Autism Spectrum Disorders
DA: Dopamine
DAT: Dopamine transporter
DBP: Dibutyl phthalate
DCHP: Dicyclohexyl phthalate
DEHP: Di(2-ethylhexyl) phthalate
GABA: γ-minobutyric acid
GD: Gestational day
Glu: Glutamate
HPLC: High-Performance Liquid Chromatography
IQ: Intelligence Quotient
PCB: Polychlorinated biphenyl
PET: Positron emission tomography
PND: Postnatal day
PtCB: 3,4,5,3′,4′-Pentachlorobiphenyl
SSRI: Selective serotonin reuptake inhibitor
TCB: 3,4,3′,4′-Tetrachlorobiphenyl

## Supplementary Files

Supplementary File 1 Supplementary (PDF, 173 KB)

## References

[1] Blumberg S.J., Bramlett M.D., Kogan M.D., Schieve L.A., Jones J.R., Lu M.C. (2013). Changes in prevalence of parent-reported autism spectrum disorder in school-aged U.S. Children: 2007 to 2011–2012. Natl. Health Stat. Rep..

[2] Brugha T.S., McManus S., Bankart J., Scott F., Purdon S., Smith J., Bebbington P., Jenkins R., Meltzer H. (2011). Epidemiology of autism spectrum disorders in adults in the community in England. Arch. Gen. Psychiatry.

[3] Baron-Cohen S., Scott F.J., Allison C., Williams J., Bolton P., Matthews F.E., Brayne C. (2009). Prevalence of autism-spectrum conditions: UK school-based population study. Br. J. Psychiatry.

[4] Nygren G., Cederlund M., Sandberg E., Gillstedt F., Arvidsson T., Carina Gillberg I., Westman Andersson G., Gillberg C. (2012). The prevalence of autism spectrum disorders in toddlers: A population study of 2-year-old swedish children. J. Autism Dev. Disord..

[5] Blaxill M.F. (2004). What’s going on? The question of time trends in autism. Public Health Rep..

[6] Wang K., Zhang H., Ma D., Bucan M., Glessner J.T., Abrahams B.S., Salyakina D., Imielinski M., Bradfield J.P., Sleiman P.M. (2009). Common genetic variants on 5p14.1 associate with autism spectrum disorders. Nature.

[7] Hallmayer J., Cleveland S., Torres A., Phillips J., Cohen B., Torigoe T., Miller J., Fedele A., Collins J., Smith K. (2011). Genetic heritability and shared environmental factors among twin pairs with autism. Arch. Gen. Psychiatry.

[8] Glessner J.T., Wang K., Cai G., Korvatska O., Kim C.E., Wood S., Zhang H., Estes A., Brune C.W., Bradfield J.P. (2009). Autism genome-wide copy number variation reveals ubiquitin and neuronal genes. Nature.

[9] Xu L.M., Li J.R., Huang Y., Zhao M., Tang X., Wei L. (2012). Autismkb: An evidence-based knowledgebase of autism genetics. Nucleic Acids Res..

[10] Stamou M., Streifel K.M., Goines P.E., Lein P.J. (2013). Neuronal connectivity as a convergent target of gene x environment interactions that confer risk for autism spectrum disorders. Neurotoxicol. Teratol..

[11] Hertz-Picciotto I., Delwiche L. (2009). The rise in autism and the role of age at diagnosis. Epidemiology.

[12] Herbert M.R. (2010). Contributions of the environment and environmentally vulnerable physiology to autism spectrum disorders. Curr. Opin. Neurol..

[13] Grandjean P., Landrigan P.J. (2006). Developmental neurotoxicity of industrial chemicals. Lancet.

[14] Wayman G.A., Bose D.D., Yang D., Lesiak A., Bruun D., Impey S., Ledoux V., Pessah I.N., Lein P.J. (2012). Pcb-95 modulates the calcium-dependent signaling pathway responsible for activity-dependent dendritic growth. Environ. Health Perspect..

[15] Schultz S.T., Klonoff-Cohen H.S., Wingard D.L., Akshoomoff N.A., Macera C.A., Ji M. (2008). Acetaminophen (paracetamol) use, measles-mumps-rubella vaccination, and autistic disorder: The results of a parent survey. Autism.

[16] Gould G.G., Seillier A., Weiss G., Giuffrida A., Burke T.F., Hensler J.G., Rock C., Tristan A., McMahon L.R., Salazar A. (2012). Acetaminophen differentially enhances social behavior and cortical cannabinoid levels in inbred mice. Prog. Neuropsychopharmacol. Biol. Psychiatry.

[17] Schultz S.T. (2010). Can autism be triggered by acetaminophen activation of the endocannabinoid system?. Acta Neurobiol. Exp. (Wars).

[18] Siniscalco D., Sapone A., Giordano C., Cirillo A., de Magistris L., Rossi F., Fasano A., Bradstreet J.J., Maione S., Antonucci N. (2013). Cannabinoid receptor type 2, but not type 1, is up-regulated in peripheral blood mononuclear cells of children affected by autistic disorders. J. Autism Dev. Disord..

[19] Bouchard M.F., Chevrier J., Harley K.G., Kogut K., Vedar M., Calderon N., Trujillo C., Johnson C., Bradman A., Barr D.B. (2011). Prenatal exposure to organophosphate pesticides and iq in 7-year-old children. Environ. Health Perspect..

[20] Engel S.M., Wetmur J., Chen J., Zhu C., Barr D.B., Canfield R.L., Wolff M.S. (2011). Prenatal exposure to organophosphates, paraoxonase 1, and cognitive development in childhood. Environ. Health Perspect..

[21] Rauh V.A., Garfinkel R., Perera F.P., Andrews H.F., Hoepner L., Barr D.B., Whitehead R., Tang D., Whyatt R.W. (2006). Impact of prenatal chlorpyrifos exposure on neurodevelopment in the first 3 years of life among inner-city children. Pediatrics.

[22] Eskenazi B., Marks A.R., Bradman A., Harley K., Barr D.B., Johnson C., Morga N., Jewell N.P. (2007). Organophosphate pesticide exposure and neurodevelopment in young Mexican-American children. Environ. Health Perspect..

[23] De Cock M., Maas Y.G., van de Bor M. (2012). Does perinatal exposure to endocrine disruptors induce autism spectrum and attention deficit hyperactivity disorders? Review. Acta Paediatr..

[24] Roberts E.M., English P.B., Grether J.K., Windham G.C., Somberg L., Wolff C. (2007). Maternal residence near agricultural pesticide applications and autism spectrum disorders among children in the California central valley. Environ. Health Perspect..

[25] Newbold R.R. (2010). Impact of environmental endocrine disrupting chemicals on the development of obesity. Hormones (Athens).

[26] Slotkin T.A. (2004). Cholinergic systems in brain development and disruption by neurotoxicants: Nicotine, environmental tobacco smoke, organophosphates. Toxicol. Appl. Pharmacol..

[27] Sunol C., Babot Z., Fonfria E., Galofre M., Garcia D., Herrera N., Iraola S., Vendrell I. (2008). Studies with neuronal cells: From basic studies of mechanisms of neurotoxicity to the prediction of chemical toxicity. Toxicol. In Vitro.

[28] Fatemi S.H., Aldinger K.A., Ashwood P., Bauman M.L., Blaha C.D., Blatt G.J., Chauhan A., Chauhan V., Dager S.R., Dickson P.E. (2012). Consensus paper: Pathological role of the cerebellum in autism. Cerebellum.

[29] Harada M., Taki M.M., Nose A., Kubo H., Mori K., Nishitani H., Matsuda T. (2011). Non-invasive evaluation of the gabaergic/glutamatergic system in autistic patients observed by mega-editing proton MR spectroscopy using a clinical 3 tesla instrument. J. Autism Dev. Disord..

[30] Hogart A., Leung K.N., Wang N.J., Wu D.J., Driscoll J., Vallero R.O., Schanen N.C., LaSalle J.M. (2009). Chromosome 15q11-13 duplication syndrome brain reveals epigenetic alterations in gene expression not predicted from copy number. J. Med. Genet..

[31] Bertone A., Hanck J., Kogan C., Chaudhuri A., Cornish K. (2010). Associating neural alterations and genotype in autism and fragile X syndrome: Incorporating perceptual phenotypes in causal modeling. J. Autism Dev. Disord..

[32] Blatt G.J., Fatemi S.H. (2011). Alterations in gabaergic biomarkers in the autism brain: Research findings and clinical implications. Anat. Rec. (Hoboken).

[33] Fatemi S.H., Reutiman T.J., Folsom T.D., Rooney R.J., Patel D.H., Thuras P.D. (2010). Mrna and protein levels for gabaaalpha4, alpha5, beta1 and gababr1 receptors are altered in brains from subjects with autism. J. Autism Dev. Disord..

[34] Jamain S., Betancur C., Quach H., Philippe A., Fellous M., Giros B., Gillberg C., Leboyer M., Bourgeron T. (2002). Linkage and association of the glutamate receptor 6 gene with autism. Mol. Psychiatry.

[35] Yang Y., Pan C. (2013). Role of metabotropic glutamate receptor 7 in autism spectrum disorders: A pilot study. Life Sci..

[36] Fatemi S.H., Halt A.R., Stary J.M., Kanodia R., Schulz S.C., Realmuto G.R. (2002). Glutamic acid decarboxylase 65 and 67 kDa proteins are reduced in autistic parietal and cerebellar cortices. Biol. Psychiatry.

[37] Yip J., Soghomonian J.J., Blatt G.J. (2007). Decreased GAD67 mrna levels in cerebellar purkinje cells in autism: Pathophysiological implications. Acta Neuropathol..

[38] Shinohe A., Hashimoto K., Nakamura K., Tsujii M., Iwata Y., Tsuchiya K.J., Sekine Y., Suda S., Suzuki K., Sugihara G. (2006). Increased serum levels of glutamate in adult patients with autism. Prog. Neuropsychopharmacol. Biol. Psychiatry.

[39] Aldred S., Moore K.M., Fitzgerald M., Waring R.H. (2003). Plasma amino acid levels in children with autism and their families. J. Autism Dev. Disord..

[40] Ballaban-Gil K., Tuchman R. (2000). Epilepsy and epileptiform EEG: Association with autism and language disorders. Ment. Retard. Dev. Disabil. Res. Rev..

[41] Sutcliffe J.S., Delahanty R.J., Prasad H.C., McCauley J.L., Han Q., Jiang L., Li C., Folstein S.E., Blakely R.D. (2005). Allelic heterogeneity at the serotonin transporter locus (slc6a4) confers susceptibility to autism and rigid-compulsive behaviors. Am. J. Hum. Genet..

[42] Levitt P. (2011). Serotonin and the autisms: A red flag or a red herring?. Arch. Gen. Psychiatry.

[43] Kumar B., Prakash A., Sewal R.K., Medhi B., Modi M. (2012). Drug therapy in autism: A present and future perspective. Pharmacol. Rep..

[44] Doyle C.A., McDougle C.J. (2012). Pharmacologic treatments for the behavioral symptoms associated with autism spectrum disorders across the lifespan. Dialogues Clin. Neurosci..

[45] Posey D.J., Kem D.L., Swiezy N.B., Sweeten T.L., Wiegand R.E., McDougle C.J. (2004). A pilot study of D-cycloserine in subjects with autistic disorder. Am. J. Psychiatry.

[46] Croen L.A., Grether J.K., Yoshida C.K., Odouli R., Hendrick V. (2011). Antidepressant use during pregnancy and childhood autism spectrum disorders. Arch. Gen. Psychiatry.

[47] Rai D., Lee B.K., Dalman C., Golding J., Lewis G., Magnusson C. (2013). Parental depression, maternal antidepressant use during pregnancy, and risk of autism spectrum disorders: Population based case-control study. BMJ.

[48] McCracken J.T., McGough J., Shah B., Cronin P., Hong D., Aman M.G., Arnold L.E., Lindsay R., Nash P., Hollway J. (2002). Risperidone in children with autism and serious behavioral problems. N. Engl. J. Med..

[49] Nakamura K., Sekine Y., Ouchi Y., Tsujii M., Yoshikawa E., Futatsubashi M., Tsuchiya K.J., Sugihara G., Iwata Y., Suzuki K. (2010). Brain serotonin and dopamine transporter bindings in adults with high-functioning autism. Arch. Gen. Psychiatry.

[50] Kaluzna-Czaplinska J., Socha E., Rynkowski J. (2010). Determination of homovanillic acid and vanillylmandelic acid in urine of autistic children by gas chromatography/mass spectrometry. Med. Sci. Monit..

[51] Hosenbocus S., Chahal R. (2012). A review of executive function deficits and pharmacological management in children and adolescents. J. Can. Acad. Child. Adolesc. Psychiatry.

[52] Happe F., Booth R., Charlton R., Hughes C. (2006). Executive function deficits in autism spectrum disorders and attention-deficit/hyperactivity disorder: Examining profiles across domains and ages. Brain Cogn..

[53] Ernst M., Zametkin A.J., Matochik J.A., Pascualvaca D., Cohen R.M. (1997). Low medial prefrontal dopaminergic activity in autistic children. Lancet.

[54] Buitelaar J.K., Willemsen-Swinkels S.H. (2000). Medication treatment in subjects with autistic spectrum disorders. Eur. Child Adolesc. Psychiatry.

[55] Fukuto T.R. (1990). Mechanism of action of organophosphorus and carbamate insecticides. Environ. Health Perspect..

[56] Soreq H., Seidman S. (2001). Acetylcholinesterase—New roles for an old actor. Nat. Rev. Neurosci..

[57] Heusinkveld H.J., Westerink R.H. (2012). Organochlorine insecticides lindane and dieldrin and their binary mixture disturb calcium homeostasis in dopaminergic pc12 cells. Environ. Sci. Technol..

[58] Silva M.H., Beauvais S.L. (2010). Human health risk assessment of endosulfan. I: Toxicology and hazard identification. Regul. Toxicol. Pharmacol..

[59] Bemis J.C., Seegal R.F. (1999). Polychlorinated biphenyls and methylmercury act synergistically to reduce rat brain dopamine content *in vitro*. Environ. Health Perspect..

[60] Bemis J.C., Seegal R.F. (2004). Pcb-induced inhibition of the vesicular monoamine transporter predicts reductions in synaptosomal dopamine content. Toxicol. Sci..

[61] Dreiem A., Okoniewski R.J., Brosch K.O., Miller V.M., Seegal R.F. (2010). Polychlorinated biphenyls and polybrominated diphenyl ethers alter striatal dopamine neurochemistry in synaptosomes from developing rats in an additive manner. Toxicol. Sci..

[62] Lyng G.D., Snyder-Keller A., Seegal R.F. (2007). Polychlorinated biphenyl-induced neurotoxicity in organotypic cocultures of developing rat ventral mesencephalon and striatum. Toxicol. Sci..

[63] Yang L., Milutinovic P.S., Brosnan R.J., Eger E.I., Sonner J.M. (2007). The plasticizer di(2-ethylhexyl) phthalate modulates gamma-aminobutyric acid type a and glycine receptor function. Anesth. Analg..

[64] Tanida T., Warita K., Ishihara K., Fukui S., Mitsuhashi T., Sugawara T., Tabuchi Y., Nanmori T., Qi W.M., Inamoto T. (2009). Fetal and neonatal exposure to three typical environmental chemicals with different mechanisms of action: Mixed exposure to phenol, phthalate, and dioxin cancels the effects of sole exposure on mouse midbrain dopaminergic nuclei. Toxicol. Lett..

[65] Cabaleiro T., Caride A., Romero A., Lafuente A. (2008). Effects of *in utero* and lactational exposure to endosulfan in prefrontal cortex of male rats. Toxicol. Lett..

[66] Dickerson S.M., Cunningham S.L., Gore A.C. (2011). Prenatal PCBs disrupt early neuroendocrine development of the rat hypothalamus. Toxicol. Appl. Pharmacol..

[67] Lafuente A., Cabaleiro T., Caride A., Gutierrez A., Esquifino A.I. (2007). Toxic effects of methoxychlor in rat striatum: Modifications in several neurotransmitters. J. Physiol. Biochem..

[68] Hilgier W., Lazarewicz J.W., Struzynska L., Frontczak-Baniewicz M., Albrecht J. (2012). Repeated exposure of adult rats to Aroclor 1254 induces neuronal injury and impairs the neurochemical manifestations of the NMDA receptor-mediated intracellular signaling in the hippocampus. Neurotoxicology.

[69] Andersen I.S., Voie O.A., Fonnum F., Mariussen E. (2009). Effects of methyl mercury in combination with polychlorinated biphenyls and brominated flame retardants on the uptake of glutamate in rat brain synaptosomes: A mathematical approach for the study of mixtures. Toxicol. Sci..

[70] Struzynska L., Sulkowski G., Dabrowska-Bouta B. (2012). Aroclor 1254 selectively inhibits expression of glial GLT-1 glutamate transporter in the forebrain of chronically exposed adult rat. Toxicology.

[71] Boix J., Cauli O., Felipo V. (2010). Developmental exposure to polychlorinated biphenyls 52, 138 or 180 affects differentially learning or motor coordination in adult rats. Mechanisms involved. Neuroscience.

[72] Boix J., Cauli O., Leslie H., Felipo V. (2011). Differential long-term effects of developmental exposure to polychlorinated biphenyls 52, 138 or 180 on motor activity and neurotransmission. Gender dependence and mechanisms involved. Neurochem. Int..

[73] Caudle W.M., Richardson J.R., Delea K.C., Guillot T.S., Wang M., Pennell K.D., Miller G.W. (2006). Polychlorinated biphenyl-induced reduction of dopamine transporter expression as a precursor to Parkinson’s disease-associated dopamine toxicity. Toxicol. Sci..

[74] Honma T., Suda M., Miyagawa M., Wang R.S., Kobayashi K., Sekiguchi S. (2009). Alteration of brain neurotransmitters in female rat offspring induced by prenatal administration of 16 and 64 mg/kg of 2,2′,4,4′,5,5′-hexachlorobiphenyl (pcb153). Ind. Health.

[75] Lafuente A., Cabaleiro T., Caride A., Esquifino A.I. (2008). Toxic effects of methoxychlor administered subcutaneously on the hypothalamic-pituitary-testicular axis in adult rats. Food Chem. Toxicol..

[76] Elnar A.A., Diesel B., Desor F., Feidt C., Bouayed J., Kiemer A.K., Soulimani R. (2012). Neurodevelopmental and behavioral toxicity via lactational exposure to the sum of six indicator non-dioxin-like-polychlorinated biphenyls (summation operator6 ndl-pcbs) in mice. Toxicology.

[77] Pourabdolhossein F., Ghasemi A., Shahroukhi A., Sherafat M.A., Khoshbaten A., Asgari A. (2009). *In vitro* assessment of paraoxon effects on GABA uptake in rat hippocampal synaptosomes. Toxicol. In Vitro.

[78] Mohammadi M., Ghani E., Ghasemi A., Khoshbaten A., Asgari A. (2008). Synaptosomal GABA uptake decreases in paraoxon-treated rat brain. Toxicology.

[79] Noriega-Ortega B.R., Armienta-Aldana E., Cervantes-Pompa J.A., Hernandez-Ruiz E., Chaparro-Huerta V., Bravo-Cuellar A., Beas-Zarate C. (2011). GABA and dopamine release from different brain regions in mice with chronic exposure to organophosphate methamidophos. J. Toxicol. Pathol..

[80] Montes de Oca L., Moreno M., Cardona D., Campa L., Sunol C., Galofre M., Flores P., Sanchez-Santed F. (2013). Long term compulsivity on the 5-choice serial reaction time task after acute chlorpyrifos exposure. Toxicol. Lett..

[81] Rush T., Liu X.Q., Hjelmhaug J., Lobner D. (2010). Mechanisms of chlorpyrifos and diazinon induced neurotoxicity in cortical culture. Neuroscience.

[82] Aldridge J.E., Meyer A., Seidler F.J., Slotkin T.A. (2005). Alterations in central nervous system serotonergic and dopaminergic synaptic activity in adulthood after prenatal or neonatal chlorpyrifos exposure. Environ. Health Perspect..

[83] Chen X.P., Wang X., Dong J.Y. (2011). Different reaction patterns of dopamine content to prenatal exposure to chlorpyrifos in different periods. J. Appl. Toxicol..

[84] Eddins D., Cerutti D., Williams P., Linney E., Levin E.D. (2010). Zebrafish provide a sensitive model of persisting neurobehavioral effects of developmental chlorpyrifos exposure: Comparison with nicotine and pilocarpine effects and relationship to dopamine deficits. Neurotoxicol. Teratol..

[85] Eells J.B., Brown T. (2009). Repeated developmental exposure to chlorpyrifos and methyl parathion causes persistent alterations in nicotinic acetylcholine subunit mRNA expression with chlorpyrifos altering dopamine metabolite levels. Neurotoxicol. Teratol..

[86] Moreno M., Canadas F., Cardona D., Sunol C., Campa L., Sanchez-Amate M.C., Flores P., Sanchez-Santed F. (2008). Long-term monoamine changes in the striatum and nucleus accumbens after acute chlorpyrifos exposure. Toxicol. Lett..

[87] Sledge D., Yen J., Morton T., Dishaw L., Petro A., Donerly S., Linney E., Levin E.D. (2011). Critical duration of exposure for developmental chlorpyrifos-induced neurobehavioral toxicity. Neurotoxicol. Teratol..

[88] Lee J.E., Park J.H., Shin I.C., Koh H.C. (2012). Reactive oxygen species regulated mitochondria-mediated apoptosis in pc12 cells exposed to chlorpyrifos. Toxicol. Appl. Pharmacol..

[89] Binukumar B.K., Bal A., Kandimalla R.J., Gill K.D. (2010). Nigrostriatal neuronal death following chronic dichlorvos exposure: Crosstalk between mitochondrial impairments, alpha synuclein aggregation, oxidative damage and behavioral changes. Mol. Brain.

[90] Binukumar B.K., Gupta N., Bal A., Gill K.D. (2011). Protection of dichlorvos induced oxidative stress and nigrostriatal neuronal death by chronic coenzyme q10 pretreatment. Toxicol. Appl. Pharmacol..

[91] Slotkin T.A., Seidler F.J. (2007). Developmental exposure to terbutaline and chlorpyrifos, separately or sequentially, elicits presynaptic serotonergic hyperactivity in juvenile and adolescent rats. Brain Res. Bull..

[92] Slotkin T.A., Seidler F.J. (2007). Prenatal chlorpyrifos exposure elicits presynaptic serotonergic and dopaminergic hyperactivity at adolescence: Critical periods for regional and sex-selective effects. Reprod. Toxicol..

[93] Xu L., Tian H., Wang W., Ru S. (2012). Effects of monocrotophos pesticide on serotonin metabolism during early development in the sea urchin, hemicentrotus pulcherrimus. Environ. Toxicol. Pharmacol..

[94] Carbone S., Samaniego Y.A., Cutrera R., Reynoso R., Cardoso N., Scacchi P., Moguilevsky J.A., Ponzo O.J. (2012). Different effects by sex on hypothalamic-pituitary axis of prepubertal offspring rats produced by in utero and lactational exposure to di-(2-ethylhexyl) phthalate (dehp). Neurotoxicology.

[95] Carbone S., Szwarcfarb B., Ponzo O., Reynoso R., Cardoso N., Deguiz L., Moguilevsky J.A., Scacchi P. (2010). Impact of gestational and lactational phthalate exposure on hypothalamic content of amino acid neurotransmitters and fsh secretion in peripubertal male rats. Neurotoxicology.

[96] Ishido M., Masuo Y., Sayato-Suzuki J., Oka S., Niki E., Morita M. (2004). Dicyclohexylphthalate causes hyperactivity in the rat concomitantly with impairment of tyrosine hydroxylase immunoreactivity. J. Neurochem..

[97] Tanaka M., DeLorey T.M., Delgado-Escueta A., Olsen R.W., Noebels J.L., Avoli M., Rogawski M.A., Olsen R.W., Delgado-Escueta A.V. (2012). Gabrb3, Epilepsy, and Neurodevelopment. Source Jasper’s Basic Mechanisms of the Epilepsies [Internet].

[98] Damodaran T.V., Patel A.G., Greenfield S.T., Dressman H.K., Lin S.M., Abou-Donia M.B. (2006). Gene expression profiles of the rat brain both immediately and 3 months following acute sarin exposure. Biochem. Pharmacol..

[99] Slotkin T.A., Ryde I.T., Levin E.D., Seidler F.J. (2008). Developmental neurotoxicity of low dose diazinon exposure of neonatal rats: Effects on serotonin systems in adolescence and adulthood. Brain Res. Bull..

[100] Richardson J.R., Caudle W.M., Wang M., Dean E.D., Pennell K.D., Miller G.W. (2006). Developmental exposure to the pesticide dieldrin alters the dopamine system and increases neurotoxicity in an animal model of parkinson’s disease. FASEB J..

[101] Padilla S., Marshall R.S., Hunter D.L., Oxendine S., Moser V.C., Southerland S.B., Mailman R.B. (2005). Neurochemical effects of chronic dietary and repeated high-level acute exposure to chlorpyrifos in rats. Toxicol. Sci..

[102] Lee D.W., Opanashuk L.A. (2004). Polychlorinated biphenyl mixture aroclor 1254-induced oxidative stress plays a role in dopaminergic cell injury. Neurotoxicology.

[103] Richardson J.R., Miller G.W. (2004). Acute exposure to Aroclor 1016 or 1260 differentially affects dopamine transporter and vesicular monoamine transporter 2 levels. Toxicol. Lett..

[104] Gerfen C.R., Miyachi S., Paletzki R., Brown P. (2002). D1 dopamine receptor supersensitivity in the dopamine-depleted striatum results from a switch in the regulation of ERK1/2/MAP kinase. J. Neurosci..

[105] Hatcher J.M., Richardson J.R., Guillot T.S., McCormack A.L., Di Monte D.A., Jones D.P., Pennell K.D., Miller G.W. (2007). Dieldrin exposure induces oxidative damage in the mouse nigrostriatal dopamine system. Exp. Neurol..

[106] Cannon J.R., Greenamyre J.T. (2011). The role of environmental exposures in neurodegeneration and neurodegenerative diseases. Toxicol. Sci..

[107] Bardin P.G., van Eeden S.F., Moolman J.A., Foden A.P., Joubert J.R. (1994). Organophosphate and carbamate poisoning. Arch. Intern. Med..

[108] Collombet J.M. (2011). Nerve agent intoxication: Recent neuropathophysiological findings and subsequent impact on medical management prospects. Toxicol. Appl. Pharmacol..

[109] Hayden K.M., Norton M.C., Darcey D., Ostbye T., Zandi P.P., Breitner J.C., Welsh-Bohmer K.A. (2010). Occupational exposure to pesticides increases the risk of incident AD: The cache county study. Neurology.

[110] Landrigan P.J., Sonawane B., Mattison D., McCally M., Garg A. (2002). Chemical contaminants in breast milk and their impacts on children’s health: An overview. Environ. Health Perspect..

[111] Seegal R.F., Brosch K.O., Okoniewski R.J. (2005). Coplanar PCB congeners increase uterine weight and frontal cortical dopamine in the developing rat: Implications for developmental neurotoxicity. Toxicol. Sci..

[112] Ghasemi A., Sadidi A., Mohammadi M., Khoshbaten A., Asgari A. (2007). Paraoxon inhibits GABA uptake in brain synaptosomes. Toxicol. In Vitro.

[113] Vandenberg L.N., Colborn T., Hayes T.B., Heindel J.J., Jacobs D.R., Lee D.H., Shioda T., Soto A.M., vom Saal F.S., Welshons W.V. (2012). Hormones and endocrine-disrupting chemicals: Low-dose effects and nonmonotonic dose responses. Endocr. Rev..

[114] Liu S.V., Schally A.V., Hawes D., Xiong S., Fazli L., Gleave M., Cai J., Groshen S., Brands F., Engel J. (2010). Expression of receptors for luteinizing hormone-releasing hormone (lh-rh) in prostate cancers following therapy with lh-rh agonists. Clin. Cancer Res..

[115] Piccart M., Parker L.M., Pritchard K.I. (2003). Oestrogen receptor downregulation: An opportunity for extending the window of endocrine therapy in advanced breast cancer. Ann. Oncol..

