# Supplementary material for: The Dynamics of Autism Spectrum Disorders: How Neurotoxic Compounds and Neurotransmitters Interact
Source: Int J Environ Res Public Health. 2013 Aug 6;10(8):3384–408. doi: 10.3390/ijerph10083384 (PMC3774444; doi:10.3390/ijerph10083384)
Supplement: Supplementary File 1 — Supplementary (PDF, 173 KB) [file ijerph-10-03384-s001.pdf]

Supplementary Information

The Dynamics of Autism Spectrum Disorders: How Neurotoxic Compounds and Neurotransmitters Interact

Abbreviations

5-HT	Serotonin	DBP	Dibutyl phthalate	LSS	Liquid scintillation spectrophotometer
A(number)	Aroclor(number)	DCHP	Dibutyl phthalate	MID	Midbrain
B_{max}	Total density receptors	DDD	Dichlorodiphenyldichloroethane	MO	Medulla oblongata
BS	Brainstem	DDE	Dichlorodiphenyldichloroethylene	NACC	Nucleus accumbens
CRBL	Cerebellum	DDT	Dichlorodiphenyltrichloroethane	NMR	Nuclear magnetic resonance
CS	Corpus striatum	DEHP	Di(2-ethylhexyl) phthalate	Ortho-TCB	2,4,2´,4´-Tetrachlorobiphenyl
CTX	Cortex	DS	Dorsal striatum	PCB	Polychlorinated biphenyl
C-	Cerebral	dpf	Days post-fertilization	PND	Post-natal day
CG-	Cingulate	DTG	Dentate gyrus	PtCB	3,4,5,3´,4´-Pentachlorobiphenyl
F-	Frontal	ELISA	Enzyme-Linked Immuno Sorbent Assay	RAR	Receptor autoradiography
M-	Motor	FB	Forebrain	RT-PCR	Real-time polymerase chain reaction
O-	Occipital	HIP	Hippocampus	SERT	Serotonin transporter
PA-	Parietal	HPLC	High Performance Liquid Chromatography	SN	Substantia nigra
PF-	Prefrontal	HTH	Hypothalamus	TCE	Trichloroethylene
TEM-	Temporal	IHC	Immunohistochemistry	TH	Tyrosine Hydroxylase
DA	Dopamine	KCI	Potassium chloride	TLCPH	Telencephalon
DAT	Dopamine Transporter	K_m	Affinity substrate enzyme	TOA	Time of analysis
VM	Ventral mesencephalon	V_{max}	Maximal initial velocity	WB	Western blot
				wpf	Weeks post-fertilization

Table S1. Literature overview organochlorines and neurotransmitters, classified by neurotransmitters. Data is presented in the following order: GABA, Glu 5-HT, DA.

Source	Method							Outcome
			Exposure			Analysis		
	Experimental group	N	Compound	Dose	Time point/Length	Location/technique	Time	
**GABA** Elnar, A. A. *et al.* , 2012 [1]	Swiss albino mice	N = 10 per treatment group	Mixture of PCB28, PCB52,PCB101, PCB138, PCB153 and PCB180	1, 10 or 100 ng/kg/day	PND0-21	RT-PCR	PND0-275 (several time points)	GABA level, gene expression $GABA_{A\alpha1}$ receptor and 5-HT1A receptor
	**Result:** No significant results							
Dickerson, S.M. *et al.*, 2011 [2]	Sprague-Dawley rats	N = 10–12 per treatment group	A1221 or mixture of PCB138, PCB153 and PCB180	1 mg/kg	GD16 and GD18	Taqman low-density arrays	PND1	Gene expression $GABA_B$ receptors 1 and 2, Glu receptors gria, gria2, gria3, grin2a, grin2b, grin2c, grin2d, vesicular Glu transporters Slc17a1, Slc17a6
	**Result:** Sign. downregulation of $GABA_B$ receptor 1 (GABBR1, $p = 0.028$), Glu receptors gria2 ($p = 0.042$) and grin2a ($p = 0.033$) and vesicular Glu transporter Slc17a1 ($p = 0.006$) in males after exposure to PCBs<br>Sign. increase of $GABA_B$ receptor 2 (GABBR2) in males after exposure to PCBs ($p < 0.001$)<br>Sign. increase of $GABA_B$ receptor 1 (GABBR1, $p < 0.001$) and Glu receptors grin2b ($p < 0.001$) in females after exposure to A1221<br>Sign. increase of $GABA_B$ receptor 1 (GABBR1, $p < 0.004$) and Glu receptor grin2c ($p = 0.03$) in females after exposure to PCBs							
Boix, J. *et al.*, 2010 [3]	Wistar rats	N = 14–33 per treatment group	PCB52, PCB138 or PCB180	1 mg/kg/day	GD7-PND21	In CRBL using microdialysis (*in vivo*)	Males 3 months post-natal, females 4 months post-natal	GABA level, Glu level
	**Result:** Sign. increase of GABA level in males and females after exposure to PCB52 ($p < 0.001$)<br>Sign. increase of Glu level in males after exposure to PCB52 ($p < 0.001$)<br>Sign. decrease of Glu level in females after exposure to PCB52 ($p < 0.001$), PCB138 ($p < 0.05$) and PCB180 ($p < 0.01$)							
Martyniuk, C.J. *et al.*, 2010 [4]	Largemouth bass	Not reported	Dieldrin	10 mg/kg	Single dose	In HIP, TLCPH and CRBL using HPLC	7 days after exposure	GABA level, DA level
	**Result:** Sign. increase of GABA level in females in CRBL and HIP after exposure to dieldrin ($p \leq 0.05$)							

Table S1. *Cont.*

Source	Method							Outcome
	Experimental group	N	Exposure			Analysis		
			Compound	Dose	Time point/Length	Location/technique	Time	
	Male Sprague-Dawley rats	N = 10 per treatment group	Endosulfan	0.61 or 6.12 mg/kg day	Gestation-weaning (PND21)	In PF-CTX using HPLC	PND15, 30, 60	GABA level, Glu level, DA level, DA turnover, 5-HT level, 5-HT turnover
Cabaleiro, T. *et al*., 2008 [5]	**Result:** Sign. increase at PND15 of Glu level ($p \leq 0.01$) after exposure to 6.12 mg/kg/day of endosulfan<br>Sign. decrease of DA turnover (DOPAC/DA) at PND15 after exposure to 0.61 mg/kg/day and 6.12 mg/kg/day of endosulfan ($p \leq 0.001$ all)<br>Sign. increase at PND30 of Glu level ($p \leq 0.05$) and 5-HT level($p \leq 0.001$) after exposure to 0.61mg/kg/day of endosulfan<br>Sign. increase at PND30 of GABA level ($p \leq 0.01$), Glu level ($p \leq 0.01$) and 5-HT level ($p \leq 0.01$) after exposure to 6.12 mg/kg/day of endosulfan<br>Sign. decrease at PND60 of GABA level ($p \leq 0.01$) and DA turnover (DOPAC/DA, $p \leq 0.05$) after exposure to 0.61 mg/kg/day of endosulfan<br>Sign. decrease at PND60 of GABA level ($p \leq 0.01$), DA turnover (DOPAC/DA, $p \leq 0.05$) and 5-HT turnover (5-HIAA/5-HT, $p \leq 0.05$) after exposure to 6.12 mg/kg/day of endosulfan<br>Sign. increase of 5-HT level at PND60 after exposure 6.12 mg/(kg day) ($p \leq 0.05$)							
Babot, Z. *et al*., 2007 [6]	Cerebellar granule cells of NRMI mice (7-days old)	Not reported	Dieldrin	3 µM	6–7 days	HPLC-F, saturation binding studies	Not reported	GABA level, Glu level, binding NMDA receptor
	**Result:** Sign. decrease of B_{max} of NMDA receptor after exposure to dieldrin ($p < 0.05$)							
Lafuente, A. *et al*., 2007 [7]	Adult male Sprague-Dawley rats	N = 10 per treatment group	Methoxychlor	25 mg/kg/day	1 month	In striatum using HPLC	Immediate freezing of striatum after exposure, TOA not reported	GABA level, Glu level, DA level, 5-HT level
	**Result:** Sign. increase of Glu level after exposure to Methoxychlor ($p \leq 0.05$)<br>Sign. increase of GABA level after exposure to Methoxychlor ($p \leq 0.001$)							
**GLUTAMATE**								
Yuk, J. *et al*., 2013 [8]	Mature Eisenia fetida earth-worms	N = 20 per treatment group	Endosulfan or Endosulfan sulfate	0.1, 1.0 or 10.0 mg/kg/day	7 days	In coelomic fluid and tissues using NMR techniques	11 days after onset exposure	Glu level
	**Result:** Sign. increase of Glu level in after exposure to 0.1, 1.0 or 10.0 mg/kg/day of endosulfan ($p < 0.05$ all)<br>Sign. increase of Glu level in after exposure to 0.1 or 10.0 mg/kg/day of endosulfan sulfate ($p < 0.05$ all)							

Table S1. *Cont.*

Source	Method							Outcome
	Experimental group	N	Exposure			Analysis		
			Compound	Dose	Time point/Length	Location/technique	Time	
Hilgier, W. *et al.*, 2012 [9]	Adult male Wistar rats	N = 4	A1254	10 mg/kg/day	14 days	Basal and HIP microdialysis, HPLC	Every 40 min in a period of 40–240 min after exposure	Glu level
	**Result:** Sign. decrease (63%) after 160 min of HIP Glu level after exposure to A1254 ($p < 0.05$)							
Strużyńska, L. *et al*, 2012 [10]	Adult male Wistar rats	N = 3–5 per treatment group	A1254	10 mg/kg/day	14 days	In CRBL and FB using WB, RT-PCR, saturation binding techniques	Immediate freezing of striatum after exposure, TOA not reported	(Gene) Expression Glu transporters EAAC1, GLT-1, GLAST, Glu uptake, Glu release
	**Result:** Sign. increase of synaptosomal Na+-dependent [3H] Glu uptake 4 and 6 minutes after addition of [3H]-Glu ($p < 0.05$)<br>Sign. increase of synaptosomal KCI-dependent [3H] Glu uptake ($p < 0.05$)<br>Sign. decrease of Na+-dependent [3H] Glu uptake in astroglial fractions of glial plasmalemmal vesicles 4, 6 and 8 minutes after addition of [3H]-Glu in the A1254 exposure group ($p < 0.05$)<br>Sign. increase of Glu transporter EAAC1 mRNA expression in FB ($p < 0.05$)<br>Sign. decrease of Glu transporter EAAC1 mRNA expression in CRBL ($p < 0.05$)<br>Sign. decrease of Glu transporter GLT-1 mRNA expression in CRBL and FB ($p < 0.05$ all)							
Boix, J. *et al.*, 2011 [11]	Wistar rats	N = 12–23 per treatment group	PCB52, PCB138 or PCB180	1 mg/kg/day	GD7-PND21	In NACC using microdialysis and HPLC	4 months of age	Glu level, DA level
	**Result:** Sign. decrease of extracellular Glu in male rats level after exposure to PCB52 ($p < 0.01$), PCB138 ($p < 0.01$) or PCB180 ($p < 0.05$)<br>Sign. decrease of extracellular Glu level in female rats after exposure to PCB52 ($p < 0.01$), PCB138 ($p < 0.05$) or PCB180 ($p < 0.05$)<br>Sign. increase of extracellular DA level in both male and female rats after exposure to PCB180 ($p < 0.05$)							
Stavenes, A. I. *et al.*, 2009 [12]	Male Wistar rats	Not reported	A1254 or PCB153	0.6–1.2 µL in 510 µL solution	15 min preincu-bation, 3 min exposure	In synaptosomes using LSS after filtration	Immediately	Glu uptake
	**Result:** Sign. inhibition of Glu uptake in a dose-dependent manner after exposure to A1254 and PCB153 ($p < 0.0001$ all)							

Table S1. *Cont.*

Source	Method							Outcome
	Experimental group	N	Exposure			Analysis		
			Compound	Dose	Time point/Length	Location/technique	Time	
**5-HT**								
Honma, T. *et al.*, 2009 [13]	Sprague-Dawley rats	N= 5–8 per treatment group	PCB153	16 or 64 mg/kg/day	GD10-16	In whole brain, FB, HB, F-CTX, O-CTX, HIP, MID, MO, striatum, CRBL, HTH using HPLC	Age of 1, 3, 6 or 9 weeks, or 1 year, dams day of weaning	5-HT level, 5-HT turnover, DA level, DA turnover
	**Result:** Sign. increase of DA level in whole brain in rats after exposure to 16 mg/kg/day after 1 week ($p < 0.05$)<br>Sign. increase of 5-HT level in whole brain in rats after exposure to 64 mg/kg/day after 1 week ($p < 0.05$)<br>Sign. decrease of DA turnover in FB and HB and of 5-HT turnover in HB in rats after exposure to 64 mg/kg/day after 9 weeks ($p < 0.05$ all)<br>Sign. decrease of DA level in O-CTX and HIP in dams after exposure to 16 mg/kg/day; and in O-CTX, HIP, MO after exposure to 64 mg/kg/day ($p < 0.05$ all)							
Lafuente, A. *et al.*, 2008 [14]	Adult male Sprague-Dawley rats	N = 10 per treatment group	Methoxychlor	25 mg/kg/day	1 month	In HTH using HPLC	Immediate freezing of brain after exposure, TOA not reported	5-HT level, 5-HT turnover
	**Result:** Sign. increase of 5-HT level in the anterior HTH after exposure to Methoxychlor ($p \leq 0.05$)<br>Sign. decrease of 5-HT level in the anterior and posterior HTH after exposure to Methoxychlor ($p \leq 0.05$ all)							
Castoldi, A.F., 2006 [15]	Sprague-Dawley rats	N = 8–13 per treatment group (per gender)	PCB153	20 mg/kg/day	GD10-16	In C-CTX, CRBL HIP, striatum using HPLC	PND21	5-HT level, DA level
	**Result:** Sign. decrease of DA level in striatum in both male and female rats ($p < 0.05$)<br>Sign. decrease of 5-HT level in C-CTX in both male and female rats ($p < 0.05$)							
Khan, I.A. *et al.*, 2004 [16]	Male Sprague-Dawley rats	N = 8–9 per treatment group	A1254	0.33 mg/g body weight	Single dose	In BS and F-CTX using HPLC	Freezing of brain 7 days after exposure, TOA not reported	5-HT level
	**Result:** Sign. decrease of 5-HT concentration in F-CTX after exposure to A1254 ($p < 0.05$)							

Table S1. *Cont.*

Source	Method							Outcome
	Experimental group	N	Exposure			Analysis		
			Compound	Dose	Time point/Length	Location/technique	Time	
**DA** Lee, D.W. *et al*., 2012 [17]	Male C57BL/6J mice	N = 20 per treatment group	A1254	6, 12 or 25 mg/kg/day	4 weeks	In striatum using HPLC, IHC	2 weeks after exposure	DA level, DA transporter (DAT) level, DA turnover
	**Result:** Sign. increase of DA level after exposure to 6 mg/kg/day ($p < 0.001$), 12 mg/kg/day ($p < 0.001$) and 25 mg/kg/day ($p < 0.05$)<br>Sign. decrease of DAT level after exposure to 6 mg/kg/day ($p < 0.05$), 12 mg/kg/day ($p < 0.01$) and 25 mg/kg/day ($p < 0.001$)							
Coccini, T. *et al*., 2011 [18]	Sprague-Dawley rats	N = 2–4 per treatment group	PCB153	5 mg/kg/day	GD7-PND21	In C-CTX and striatum using saturation binding techniques	PND21 or 36	Density and affinity of DA receptors D_1 and D_2,
	**Result:** Sign. decrease at PND21 of D_1 density in the striatum and C-CTX in male rats ($p < 0.05$ all)<br>Sign. increase of D_2 density in the cortex at PND21 in male rats and at PND36 in male and female rats ($p < 0.05$ all)<br>Sign. decrease of D_2 affinity in the cerebral cortex at PND21 in male rats and at PND36 in male and female rats ($p < 0.05$ all)							
Tian, Y.H. *et al*. (2011) [19]	ICR mice	N = 18–34 per treatment group	A1254	6 or 18 mg/kg/day	LD 7–21 (exposure mothers) and PND22-42	In striatum (D1 and D2, DAT) and F-CTX, CG-CTX, MCTX, DS, HIP and DTG (NMDA) using RAR	Immediate freezing of brain after exposure, TOA not reported	Affinity of DA receptors D_1 and D_2, DA transporter (DAT), binding NMDA receptor
	**Result:** Sign. decrease of NMDA receptor binding in F-CTX ($p < 0.05$), C-CTX ($p < 0.05$), M-CTX ($p < 0.01$), DS, ($p < 0.05$), HIP (CA3, $p < 0.01$) and DT ($p < 0.05$) after exposure to 18 mg/kg/day							
Dreiem, A. *et al*., 2010 [20]	Long-Evans rats	N = 5–12 rats, per treatment group	Mixture of A1242, A1248, A1254 and A1260	10, 20 or 40 µM	Single dose on PND7, 14 or 21	In medium (extraneuronal) and synaptosomes (striatal tissue) using HPLC	Immediate freezing of brain after exposure, TOA not reported	DA level
	**Result:** Sign. decrease of synaptosomal DA level at PND7 ($p \leq 0.01$), PND14 ($p \leq 0.001$) and PND21($p \leq 0.05$) after exposure to 20 µM; and at PND7 ($p \leq 0.001$), PND14 ($p \leq 0.001$) and PND21 ($p \leq 0.001$) after exposure to 40 µM<br>Sign. interaction effect between exposure and age ($p \leq 0.01$), with a decrease of synaptosomal DA level<br>Sign. increase of extraneuronal (medium) DA level at PND7 ($p \leq 0.001$), PND14 ($p \leq 0.01$) and PND21($p \leq 0.01$) after exposure to 10 µM; at PND7 ($p \leq 0.001$), PND14 ($p \leq 0.001$) and PND21 ($p \leq 0.001$) after exposure to 20 µM; and at PND7 ($p \leq 0.001$), PND14 ($p \leq 0.001$) and PND21($p \leq 0.001$) after exposure to 40 µM<br>Sign. increase of synaptosomal DA level on PND14 and PND21, compared to PND7 ($p \leq 0.001$)<br>Sign. decrease ofextraneuronal (medium) DA level on PND14 and PND21, compared to PND7 ($p \leq 0.001$)							

Table S1. *Cont.*

Source	Method							Outcome
	Experimental group	N	Exposure			Analysis		
			Compound	Dose	Time point/Length	Location/technique	Time	
Faro, L.R. *et al*., 2009 [21]	Adult female Sprague-Dawley rats (*in vivo*)	N = 5–6 per treatment group	DDT, lindane, dicofol	1 mM	60 min	In striatum (extracell.) using microdialysis and HPLC	2 h after exposure	DA release
	Sign. increase of DA level 80 min after initiation of exposure to DDT ($p < 0.01$) and lindane ($p < 0.05$)							
Schuh, R.A. *et al*., 2009 [22]	Female CD-1 mice	Not reported	Methoxychlor	16, 32 or 64 mg/kg/day	20 days	In striatum using HPLC, WB	Freezing of brain within 24 h after exposure, TOA not reported	DA level, DA transporter (DAT), DA turnover, 5-HT level (5-HT)
	**Result:** Sign. decrease of DA level after exposure to 64 mg/kg/day ($p < 0.05$)<br>Sign. decrease of DAT level after exposure to 16, 32 and 64 mg/kg/day ($p < 0.05$)<br>Sign. decrease of DA turnover after exposure to 16 mg/kg/day ($p < 0.05$) and 32 mg/kg/day ($p < 0.05$)							
Gash, D.M. *et al*., 2008 [23]	Adult male Fischer 344 rats	N = 17 per treatment group	TCE	1,000 mg/kg/day	5 days a week for 6 weeks	In striatum, SN using HPLC–EC	Not reported	DA level
	**Result:** Sign. decrease of DA level in SN ($p < 0.05$)							
Hatcher, J.M. *et al*., 2008 [24]	Male C57BL/6J mice	N = 6 per treatment group (N = 12 controls)	DDT, DDE, DDD	Initial exposure of DDT 1 or 3 mg/kg/3 days, DDE 1, 3 or 6 mg/kg/3 days, second exposure of 1, 10 or 100 µM of DDT, DDE, DDD or dieldrin	Initial exposure: 30 days (every 3 days) Second exposure: single dose	Cellular, vesicular and synaptosomal analysis using filtration, saturation binding techniques, HPLC, WB	3 days after treatment	DA level, DA uptake, DA release
	**Result:** Sign. decrease of DAT-mediated ^{3}H-DA uptake after exposure to 1, 10, or 100 µM of DDD; 10 or 100 µM of DDT; 100 µM of DDE or 100 µM of dieldrin ($p < 0.05$ all)<br>Sign. decrease of ^{3}H-DA release from isolated vesicles after exposure to 100 µM of DDD ($p < 0.05$)<br>Sign. decrease of synaptosomal ^{3}H-DA release after exposure to 10 or 100 µM of DDD; 100 µM of DDT; 100 µM of DDE or 1 or 100 µM of dieldrin ($p < 0.05$ all)							

Table S1. *Cont.*

Source	Method							Outcome
	Experimental group	N	Exposure			Analysis		
			Compound	Dose	Time point/Length	Location/technique	Time	
Hatcher, J.M. *et al*., 2007 [24]	Male C57BL/6J mice	N = 12 per treatment group	Dieldrin	0.3 mg/kg, 1 mg/kg or 3 mg/kg	Every 3 days for 30 days	In striatum using HPLC, filtration, WB	3 days after end exposure	DA level, DA turnover, DAT level, DAT mRNA , DA uptake, DAT binding
	**Result:** Sign. decrease of DAT level after exposure to 3 mg/kg (26.9%, $p < 0.05$) of dieldrin<br>Sign. decrease of DA turnover (DOPAC/DA) after exposure to 1 mg/kg (32.5%, $p < 0.01$) and 3 mg/kg (23.5%, $p < 0.05$) of dieldrin<br>Sign. decrease of DAT binding sites after exposure to 1 mg/kg (12.4%, $p < 0.01$) and 3 mg/kg (14.7%, $p < 0.01$) of dieldrin<br>Sign. decrease of DAT-mediated ^{3}H-DA uptake after exposure to 3 mg/kg (21.2%, $p < 0.05$) of dieldrin							
Lyng G.D. *et al*., 2007 [25]	Co-cultures of Fetal Sprague-Dawley rats	Not reported	PCB mixture of 35% A1242, 35% A1248, 15% A1254, 15% A1260	2 µM or 8 µM	1, 3, 7, or 14 days	In co-cult. of VM, striatum (later separated) and extraneuronal (medium) using HPLC, TH IHC, fluoro jade B labeling, WB	Immediately	DA level, DA neurons (VM), DAT level
	**Result:** Sign. decrease of DA level in striatum and VM after exposure to 8 µM for 1 day, 3 days, 7 days or 14 days ($p \leq 0.05$ all)<br>Sign. decrease of DA level in striatum after exposure to 2 µM for 7 days($p \leq 0.001$); and in VM after exposure to 2 µM for 14 days ($p \leq 0.05$)<br>Sign. increase of extraneuronal (medium) DA level after exposure to 8 µM for 1 day ($p \leq 0.001$) and 7 days ($p \leq 0.01$)<br>Sign. decrease of DA neurons after exposure to 2 µM for 14 days ($p \leq 0.01$); and to 8 µM for 7 days (28%, $p \leq 0.01$) and 14 days (48%, $p \leq 0.01$)<br>Sign. decrease of DAT protein level in striatum after exposure to 2 µM for 3 days (16% decrease, $p \leq 0.05$), 7 days (39% decrease, $p \leq 0.01$) and 14 days (35% decrease, $p \leq 0.05$); and 8 µM for 3 days (36% decrease, $p \leq 0.001$), 7 days (57% decrease, $p \leq 0.001$) and 14 days (62% decrease, $p \leq 0.01$)<br>Sign. decrease of DAT protein level in VM after exposure to 2 µM for 7 days (58% decrease, $p \leq 0.01$) and 14 days (33% decrease, $p \leq 0.05$); and to 8 µM for 7 days (77% decrease, $p \leq 0.05$) and 14 days (64% decrease, $p \leq 0.01$)							
Caudle, W. M. *et al*., 2006 [26]	Adult C57BL/6J mice	Not reported	Mixture of A1254 and A1260	7.5 or 15 mg/kg/day	3, 7, 14 or 30 days	In striatum and VM (DAT mRNA only) using saturation binding techniques, HPLC, WB , RT-PCR	24 h after exposure	DA level,<br>DA uptake,<br>DAT binding<br>DAT level,<br>DAT mRNA , SERT binding
	**Result:** Sign. decrease of striatal DAT level after exposure to 7.5 mg/kg/day after 30 days ($p < 0.01$); and to 15 mg/day after 14 days ($p < 0.01$) and 30 days ($p < 0.001$)<br>Sign. decrease of ^{3}H-DA uptake and ^{3}H-DA binding after exposure to 7.5 mg/kg/day after 14 days ($p < 0.05$ all) and 30 days ($p < 0.001$ all)<br>Sign. decrease of ^{3}H-DA uptake and ^{3}H-DA binding after exposure to 15 mg/kg/day after 14 days ($p < 0.001$ all) and 30 days ($p < 0.001$ all)							

Table S1. *Cont.*

Source	Method							Outcome
	Experimental group	N	Exposure			Analysis		
			Compound	Dose	Time point/Length	Location/technique	Time	
Richardson, J. R., 2006 [27]	C57BL/6J mice	Not reported	Dieldrin	0.3, 1 or 3 mg/kg/3 days	Every 3 days for 2 weeks	In striatum and VM (RNA only) using HPLC, WB, RT-PCR	PND22	DA level, DA turnover, DAT level, DAT mRNA , 5-HT (5-HT) transporter level, GABA transporter level
	**Result:** Sign. increase of DAT level in both males and females after exposure to 0.3 ($p < 0.01$), 1 ($p < 0.001$) and 3 mg/kg ($p < 0.001$) of dieldrin<br>Sign. increase of DAT mRNA level in males after exposure to 1 mg/kg ($p < 0.05$) and 3 mg/kg ($p < 0.01$) of dieldrin<br>Sign. increase of DA turnover (DOPAC/DA) in males after exposure to 0.3 ($p < 0.001$), 1 ($p < 0.001$) and 3 mg/kg ($p < 0.001$) of dieldrin							
Vettori, M.V. *et al*., 2006 [28]	PC12 cell cultures	Not reported	PCB153	0,<br>1e−5, 5e−5, 1e−4, 2e−4, 4e−4 M	Single dose	HPLC	24 h after exposure	DA level
	**Result:** Sign. decrease of intracellular DA levels after exposure to 2e-4 ($p < 0.01$) of PCB153							
Jelaso, A.M. *et al*., 2005 [29]	Xenopus laevis tadpoles	N = 12 per treatment group	A1254	24, 50, 100, or 200 ppm	Stage 44/ 45–64/65	RT-PCR	Immediate freezing of embryo after exposure, TOA not reported	Gene expression DA type 2 receptor (D2R)
	**Result:** No significant results							
Seegal, R.F. *et al*., 2005 [30]	Sprague-Dawley rats	N = 2 per treatment group	PtCB, TCB or ortho-TCB	PtCB 0.25 or 1 µg/kg/day, TCB 0.1 or 1 mg/kg/day and ortho-TCB 1, 10 or 20 mg/kg/day	GD6 - PND21	In PF-CTX using HPLC	Freezing of brain at PND35, 60 or 90, TOA not reported	DA level
	Result: Sign. increase of DA level after exposure to 0.1 mg/kg of TCB after 90 days ($p \leq 0.05$); to 1 mg/kg of TCB after 35 days ($p \leq 0.05$), 60 days ($p \leq 0.01$) and 90 days ($p \leq 0.001$); and to 1 µg/kg of PtCB after 35 days ($p \leq 0.01$) and 90 days ($p \leq 0.001$)<br>Sign. decrease of DA level after exposure to 20 mg/kg of ortho-TCB after 35 days ($p \leq 0.001$) and 60 days ($p \leq 0.01$)							
Bemis, J.C. *et al*., 2004 [31]	Long-Evans rats	N = 9–18 per treatment group	PCB77, PCB91, PCB95, PCB103, PCB126, PCB153 or A1254	2.5, 5, 10, 20, or 40 µM	30 min	In striatal synaptosomes and extraneuronal (medium) using HPLC	1–3 days after exposure	DA level
	**Result:** Sign. decrease of synaptosomal DA level after exposure to 2.5 µM ($p \leq 0.05$), 5 µM ($p \leq 0.001$), 10 µM ($p \leq 0.001$), 20 µM ($p \leq 0.001$) and 40 µM ($p \leq 0.001$) of PCB95; to 20 µM ($p \leq 0.05$) and 40 µM ($p \leq 0.01$) of PCB153; to 10 µM ($p \leq 0.05$), 20 µM ($p \leq 0.001$) or 40 µM ($p \leq 0.001$) of PCB91; or to 40 µM of PCB103 or A1254 ($p \leq 0.001$ both)<br>Sign. decrease of extraneuronal (medium) DA level after exposure to 20 µM ($p \leq 0.001$) and 40 µM ($p \leq 0.01$) of PCB95; to 20 µM ($p \leq 0.05$) and 40 µM ($p \leq 0.001$) of PCB91; to 20 µM ($p \leq 0.001$) and 40 µM ($p \leq 0.001$) of PCB103; or to 5 µM ($p \leq 0.05$), 10 µM ($p \leq 0.001$), 20 µM ($p \leq 0.001$) and 40 µM ($p \leq 0.001$) of A1254							

Table S1. *Cont.*

**Source**	**Method**							**Outcome**
	**Experimental group**	**N**	**Exposure**			**Analysis**		
			**Compound**	**Dose**	**Time point/Length**	**Location/technique**	**Time**	
Kang, J.H. *et al*., 2004 [16]	Catechola-minergic cells	Not reported	A1254	10 µg/mL	Single dose	Intracellular analysis using HPLC	3 h	DA level
	**Result:** Sign. decrease of DA level after exposure to A1254($p < 0.001$)							
Lee, D.W. *et al*., 2004 [32]	MN9D cells	Not reported	A1254	1, 5, 10, or 20 ppm	3, 12, 24 or 48 h	HPLC	Not reported	DA level, DA turnover
	**Result:** Sign. decrease of DA level after 24 h exposure to 1 ppm ($p < 0.05$), 5 ppm ($p < 0.0001$), 10 ppm ($p < 0.0001$) and 20 ppm ($p < 0.0001$) of A1254<br>Sign. increase of DA turnover after 24 h exposure to 20 ppm ($p < 0.05$) of A1254							
Richardson, J.R. *et al*., 2004 [33]	C57BL/6J mice	Not reported	A1016 or A1260	500 mg/kg	Single dose	In striatum using HPLC, WB	Freezing of brain 1, 7 or 14 days after exposure, TOA not reported	DA level, DAT level, DA turnover
	**Result:** Sign. decrease of DA level after exposure to A1016 after 1 day ($p \leq 0.05$), 7 days ($p \leq 0.01$) and 14 days ($p \leq 0.001$); or to A1260 after 1 day ($p \leq 0.05$), 7 days ($p \leq 0.001$) and 14 days ($p \leq 0.001$)<br>Sign. decrease of DAT level after exposure to A1260 after 1 day ($p \leq 0.005$) and 7 days ($p \leq 0.0001$); or to A1016 after 1 day ($p \leq 0.005$), 7 days ($p \leq 0.0001$) and 14 days ($p \leq 0.01$)<br>Sign. increase of DA turnover after exposure to A1016 after 7 days ($p \leq 0.05$) and 14 days ($p \leq 0.05$); or to A1260 after 7 days and 14 days ($p \leq 0.05$ both)							

Table S2. Literature overview organophosphates and neurotransmitters, classified by neurotransmitters. Data is presented in the following order: GABA, Glu, 5-HT, DA.

Source	Method							Outcome
	Population	N	Exposure			Analysis		
	Experimental group		Compound	Dose	Period	Location/ technique	Time	
**GABA**								
Montes de Oca, L. *et al*., 2013 [34]	Adult male Lister Hooded rats	Not reported	Chlorpyrifos	250 mg/kg	Single dose	In striatum and HIP using HPLC	15 months after exposure	GABA level, Glu level
	**Result:** Sign. decrease of GABA level after exposure to chlorpyrifos ($p \leq 0.05$)<br>Sign. decrease of Glu level after exposure to chlorpyrifos ($p \leq 0.05$)							
Noriega-Ortega, B.R. *et al*., 2011 [35]	Male BALB/c mice	N = 20 per treatment group	Methamido-phos	2.6 mg/kg	Every 3 days	In C-CTX, striatum and HIP using filtration	3, 6 and 9 months, using fractions of 10 min	GABA release, DA release
	**Result:** Sign. increase of GABA release in C-CTX after 9 months (fraction 7; $p < 0.001$ and fraction 8; $p < 0.001$) of exposure to methamidophos<br>Sign. decrease of GABA release in C-CTX after 3 months (fraction 7; $p < 0.001$ and fraction 8; $p < 0.05$) and 6 months (fraction 5; $p < 0.001$, fraction 6; $p < 0.001$, fraction 7; $p < 0.001$ and fraction 8; $p < 0.001$) of exposure to methamidophos<br>Sign. decrease of GABA release in HIP after 3 months (fraction 7; $p < 0.001$ and fraction 7; $p < 0.001$) and 6 months (fraction 7; $p < 0.001$ and fraction 8; $p < 0.05$) of exposure to methamidophos							
Pourabdol-hossein, F. *et al*., 2009 [36]	Male Wistar rats	Not reported	Paraoxon	0, 0.01, 0.1, 1, 10 or 100 µM	10 min	In hippocampal synaptosomes using LSS after filtration	Immediately	GABA uptake
	**Result:** Sign. decrease of GABA uptake after exposure to 0.1 µM ($p < 0.05$) and 1 µM ($p < 0.05$) of paraoxon							
Mohammadi, M. *et al*., 2008 [37]	Adult male Wistar rats	N = 5–7 per treatment group	Paraoxon	0.1, 0.3 or 0.7 mg/kg	Single dose	In hippocampal and C-CTX synaptosomes using LSS after filtration	30 min, 4 or 8 h after exposure	GABA uptake
	**Result:** Sign. decrease of GABA uptake after exposure to 0.1, 0.3 and 0.7 mg/kg of paraoxon ($p < 0.001$ all)							
Shahroukhi, A. *et al*., 2007 [38]	Adult male Wistar rats	N = 73 (total)	Paraoxon	0, 0.01, 0.1, 1, 10 or 100 µM	20 min	In cerebellar synaptosomes using LSS after filtration	Immediately	GABA uptake
	**Result:** Sign. decrease of GABA uptake after exposure to 1, 10 or 100 µM of paraoxon ($p < 0.05$ all)							

Table S2. *Cont.*

Source	Method							Outcome
	Population	N	Exposure			Analysis		
	Experimental group		Compound	Dose	Period	Location/ technique	Time	
Ghasemi, A. *et al.*, 2007 [39]	Male Wistar rats	Not reported	Paraoxon	10^{-9}–10^{-3} M	20 min	In C-CTX synaptosomes using LSS after filtration	Immediately	GABA uptake, K_m and V_{max} (kinetic)
	**Result:** Sign. increase of GABA uptake after exposure to 10^{-8}, 10^{-7}, 10^{-6} of paraoxon ($p < 0.01$ for all)<br>Sign. decrease of GABA uptake after exposure to 10^{-3} of paraoxon ($p < 0.05$)<br>Sign. overall decrease of V_{max} after exposure to paraoxon ($p < 0.001$)							
**GLUTAMATE**<br>Rush, T. *et al.*, 2010 [40]	Cortical cell cult. Swiss Webster mice	N = 8–16	Chlorpyrifos or Diazinon	CPF: 100 µM,<br>DZN: 30 µM	6 h	In pure and mixed neuronal/ glial cultures using HPLC	Not reported	Glu level
	**Result:** Sign. increase of extracellular Glu level in mixed neuronal and glial cultures, pure neuronal and pure glial cultures after exposure to chlorpyrifos ($p < 0.05$)							
**5-HT**<br>Xu, L. *et al.*, 2012 [41]	Embryo sea urchins (H. pulcherrimus)	N = 3–12 per treatment group	Monocrotophos	0.01, 0.10 or 1.00 mg/L	12–48 h	In embryo/larvae using RT-PCR and ELISA	Immediate freezing of worms at 12-hpf, 15-hpf, 18-hpf, 24-hpf, 30-hpf, 36-hpf, or 48-hpf, TOA not reported	5-HT level, 5-HT reuptake transporter level (mRNA)
	**Result:** Sign. increase of SERT mRNA expression at the 24-hpf stage after exposure to 0.01 mg/L of monocrotophos ($p < 0.05$); and at the 30-hpf and 36-hpf stage after exposure to 0.01, 0.10 and 1.00 mg/L of monocrotophos ($p < 0.05$ all)<br>Sign. decrease of SERT mRNA expression at the 36-hpf stage and the 48-hpf stage after exposure to 1.00 mg/L of monocrotophos ($p < 0.05$ all)<br>Sign. decrease of 5-HT level at the 18-hpf stage after exposure to 0.01 mg/L of monocrotophos ($p < 0.05$); and at the 48-hpf stage after exposure to 0.10 ($p < 0.05$) or 1.00 mg/L of monocrotophos ($p < 0.05$)<br>Sign. increase of 5-HT level at the 30-hpf stage after exposure to 1.00 mg/L of monocrotophos ($p < 0.05$); and at the 36-hpf stage after exposure to 0.01 ($p < 0.05$) or 0.10 mg/L of monocrotophos ($p < 0.01$)							
Eddins, D. *et al.*, 2010 [42]	Zebrafish	N = 30–50 per treatment group	Chlorpyrifos	0.29 µM	2 hpf–5 dpf	HPLC	6 dpf, 22 wpf	5-HT level, 5-HT turnover, DA level, DA turnover
	**Result:** Sign. decrease of DA level and 5-HT level in 6 day old zebrafish larvae after exposure to chlorpyrifos ($p < 0.01$ all)<br>Sign. increase of DA turnover in 6 day old zebrafish larvae after exposure to chlorpyrifos ($p < 0.025$)<br>Sign. decrease of DA level in adult zebrafish after exposure to chlorpyrifos ($p < 0.025$)							

Table S2. *Cont.*

Source	Method							Outcome
	Population	N	Exposure			Analysis		
	Experimental group		Compound	Dose	Period	Location/ technique	Time	
Moreno, M., 2008 [43]	Male Wistar rats	N = 5–12 per treatment group	Chlorpyrifos	250 mg/kg	Single dose	In striatum and NACC using HPLC	2, 7, 15 or 30 days after exposure	5-HT level, 5-HT turnover, DA level, DA turnover
	**Result:** Sign. increase of DA turnover (DOPAC/DA ratio) in the striatum 2 days after exposure to chlorpyrifos ($p < 0.05$)<br>Sign. decrease of 5-HT turnover (5-HIAA/5-HT ratio) in the striatum 7 and 15 days after exposure to chlorpyrifos ($p < 0.05$ all)<br>Sign. decrease of DA level and 5-HT level in the NACC 30 days after exposure to chlorpyrifos ($p < 0.05$ all)							
Slotkin, T.A., *et al*., 2008 [44]	Sprague-Dawley rats	N = 6 per treatment group	Diazinon	0.5 or 2 mg/kg/day	PND1-4	In F/PA-CTX, TEM/OC-CTX, BS using saturation binding techniques	Freezing of brain at PND30, 60 or 100, TOA not reported	Binding 5-HT receptors $5HT_{1A}$ and $5HT_2$, 5-HT (5HT) transporter
	**Result:** Sign. decrease of $5HT_{1A}$ receptors in male rats exposed to 0.5 mg/kg of diazinon ($p < 0.05$)<br>Sign. increase of $5HT_{1A}$ transporters in female rats exposed to 0.5 mg/kg of diazinon ($p < 0.05$)							
Slotkin, T.A. *et al*., 2007 [45]	Sprague-Dawley rats	N = 8–12 per treatment group	Chlorpyrifos	1 or 5 mg/kg/day	GD9–12 or GD17–20	In striatum, HIP, C-CTX, MID and BS using HPLC	Freezing of brain at PND30, TOA not reported	5-HT level, 5-HT turnover, DA level, DA turnover
	**Result:** Significant main treatment effect (increasing) on 5-HT level in the striatum after exposure from GD17-20 to chlorpyrifos ($p < 0.03$)							
Slotkin, T.A. *et al*., 2007 [46]	Sprague-Dawley rats	N = 12 per treatment group	Chlorpyrifos	5 mg/kg/day	PND11–14	In C-CTX, HIP, MID and BS using HPLC	PND21, 30 or 45, TOA not reported	5-HT level, 5-HT turnover
	**Result:** Sign. decrease at PND21 of 5-HT level in BS after exposure to chlorpyrifos ($p < 0.05$)<br>Sign. increase at PND21 of 5-HT turnover in BS after exposure to chlorpyrifos ($p < 0.05$)							

Table S2. *Cont.*

Source	Method							Outcome
	Population	N	Exposure			Analysis		
	Experimental group		Compound	Dose	Period	Location/ technique	Time	
Aldridge, J.E. *et al.*, 2005 [47]	Sprague-Dawley rats	N = 12 per treatment group	Chlorpyrifos	GD17–20: 1 or 5 mg/kg/day, PND1–4: 1 mg/kg/day, PND11–14: 5 mg/kg/day	GD17–20, PND1–4, PND11–14	In striatum, C-CTX, HIP, MID and BS using HPLC	Freezing of brain at PND60, TOA not reported	5-HT level, 5-HT turnover, DA level (only at GD17–20), DA turnover
	**Result:** Sign. effect on 5-HT level after exposure to 5 mg/kg of chlorpyrifos during GD17-20 ($p < 0.03$)<br>Sign. effect on 5-HT turnover ($p < 0.007$) after exposure to 1 mg/kg ($p < 0.05$) and 5 mg/kg chlorpyrifos ($p < 0.002$) during GD17–20<br>Sign. decrease of hippocampal DA level after exposure to 1 mg/kg ($p < 0.04$) and 5 mg/kg of chlorpyrifos during GD17–20 ($p < 0.04$)<br>Sign. increase of DA turnover in the cerebral cortex, stratum and midbrain after exposure to 5 mg/kg of chlorpyrifos during GD17–20 ($p < 0.02$)<br>Sign. overall decrease of 5-HT level in females after exposure to 1 mg/kg chlorpyrifos during PND1–4 ($p < 0.03$)							
**DA**								
Lee, J.E. *et al.*, 2012 [17]	PC12 cell cult. adult female Sprague-Dawley rats	Not reported	Chlorpyrifos	100 µM	12 h	Intracellular and extraneuronal (medium) using HPLC	Not reported	DA level
	**Result:** Sign. decrease of intracellular DA level after exposure to chlorpyrifos ($p < 0.01$)<br>Sign. decrease of extraneuronal (medium) DA level after exposure to chlorpyrifos ($p < 0.01$)							
Binukumar, B.K. *et al.*, 2011 [48]	Male Wistar rats	N = 10 per treatment group	Dichlorvos	2.50 mg/kg/day	12 weeks	In SN and CS using HPLC	Not reported	DA level
	**Result:** Sign. decrease of DA level in SN and CS after exposure to dichlorvos ($p < 0.001$)							
Chen, X.P. *et al.*, 2011 [49]	ICR mice	N = 6 per treatment group	Chlorpyrifos	5 mg/kg^{-1}/day	GD7.5–11.5 or GD13–17	In C-CTX and HIP using fluorescence techniques	Freezing of C-CTX at GD17, PND14, and PND60, freezing of HIP at PND14 and PND60, TOA not reported	DA level
	**Result:** Sign. decrease of DA level in C-CTX at GD17 ($p < 0.01$) and PND14 ($p < 0.05$) after exposure to chlorpyrifos during GD7.5–11.5; and at PND14 ($p < 0.01$) and PND60 ($p < 0.01$) after exposure to chlorpyrifos during GD13–17<br>Sign. decrease of DA level in the HIP at PND14 ($p < 0.01$) and PND60 ($p < 0.01$) after exposure to chlorpyrifos during GD7.5-11.5							

Table S2. *Cont.*

Source	Method							Outcome
	Population	N	Exposure			Analysis		
	Experimental group		Compound	Dose	Period	Location/ technique	Time	
Sledge, D. *et al*., 2011 [50]	Zebrafish embryos	N = 18–34 per group	Chlorpyrifos	100 ng/mL	dpf 1, 1–2, 1–3, 1–4 or 1–5	In whole brain using HPLC	Not reported	DA level, 5-HT level
	**Result:** Sign. decreasing linear duration effect of exposure to chlorpyrifos on DA level ($p < 0.025$)<br>Sign. decrease of DA level after exposure to chorpyrifos during dpf 1–5 ($p < 0.05$)							
Binukumar, B.K. *et al*., 2010 [51]	Male Wistar rats	N = 6 per treatment group	Dichlorvos	2.5 mg/kg/day	12 weeks	In SN and CS using HPLC	Not reported	DA level
	**Result:** Sign. decrease of DA level in SN and CS after exposure to dichlorvos ($p < 0.001$)							
Eells, J.B. *et al*., 2009 [52]	Sprague-Dawley rats	Not reported	Chlorpyrifos, Methyl parathion	CPF: 1.5 mg PND1–7, 3 mg/kg/day PND8–14, 6 mg/kg/day PND15–21, MPT: 0.3 mg/kg/day PND1–7, 0.6 mg/kg/day PND8–14, 0.9 mg/kg/day PND15–21	PND1–21	In striatum using HPLC and RT-PCR	Freezing of brain at PND22 or PND50, TOA not reported	DA level, DA turnover, gene expression DAT
	**Result:** Sign. increase at PND50 of DA (DOPAC/DA) turnover after exposure to chlorpyrifos ($p < 0.05$)							
Damodaran, T.V. *et al*., 2006 [53]	Adult male Sprague-Dawley rats	Not reported	Sarin	50 or 100 µg/kg	Single dose	In whole brain using several techniques, including microarray and RT-PCR	Freezing of brain 15 min (50 µg/kg) or 3 months (100 µg/kg) after exposure, TOA not reported	Gene expression all genes (no selection)
	**Result:** Sign. upregulation of $GABA_B$ receptor 1d (Gabbr-1), $GABA_A$ receptor alpha-1 subunit (Gabra1), DRd4, 5-HT receptor (Htr6) and 5-HT neurotransmitter transporter (Slc6a4) after 50 µg/kg of exposure to sarin ($p < 0.05$ all)<br>Sign. upregulation of $GABA_B$ receptor 1d (Gabbr-1), $GABA_A$ receptor alpha-3 subunit (Gabra3), $GABA_A$ receptor beta-3 subunit (Gabrb3) and Glu receptor (Gria1) after 100 µg/kg of exposure to sarin ($p < 0.05$ all)							
Padilla, S. *et al*., 2005 [54]	Male Long-Evans rats	N = 5–8 per treatment group	Chlorpyrifos	0, 1, or 5 mg/kg/day, additional spike doses of 60 mg/kg (2 months) and 45 mg/kg (4, 6, 8, 10, 12 months)	6 or 12 months	In striatum using incubation and filtration	Freezing of brain at 6, 12 or 15 months, TOA not reported	DA level, DAT density
	**Result:** Significant increase of DAT density 24 hours after exposure to a spike after 6 months of exposure to 0 mg/kg/day ($p < 0.05$), 1 mg/kg/day ($p < 0.05$) or 5 mg/kg/day ($p < 0.05$) of chlorpyrifos							

Table S3. Literature overview phthalates and neurotransmitters, classified by neurotransmitters. Data is presented in the following order: GABA, Glu, 5-HT, DA.

Source	Method							Outcome
	Population	N	Exposure			Analysis		
	Experimental group		Compound	Dose	Length	Location/ Technique	Time	
**GABA** Carbone, S. *et al*., 2012 [55]	Wistar rats	N = 8–10	DEHP	3 or 30 mg/kg/day	GD1-weaning	In HIP using HPLC	Freezing at PND30, TOA not reported	GABA level
	**Result:** Sign. increase of GABA level in peripubertal male rats after exposure to 30 mg/kg of DEHP ($p < 0.01$)							
Carbone, S. *et al*., 2010 [56]	Wistar male rats	N = 10	DEHP	3 or 30 mg/kg/day	GD1-weaning	In HIP using HPLC	Freezing at PND15, TOA not reported	GABA level, Glu level
	**Result:** Sign. decrease of GABA level in prepubertal male rats after exposure to 30 mg/kg of DEHP ($p < 0.01$)<br>Sign. increase of GABA level in prepubertal female rats after exposure to 30 mg/kg of DEHP ($p < 0.01$)							
**GLUTAMATE 5-HT DA** Ishido, M. *et al*., 2004 [57]	Male Wistar rats	Not reported	DCHP	29 µg	Single dose (PND5)	In striatum and MID using macroarray	Freezing at 4 and 8 weeks of age, TOA not reported	Gene expression of: GABA transporters/receptors, Glu transporters/ receptors, DA transporters/receptors, 5-HT transporters/ receptors
	**Result:** Upregulation of ionotropic Glu receptor epsilon 2 (ratio 1.60); metabotropic Glu receptor 1 (ratio 3.50); metabotropic Glu receptor 4 (ratio 1.86); metabotropic Glu receptor 7 (ratio 1.80); Glu transporter (ratio 4.50); GABA-A receptor alpha 1 (ratio 2.50); GABA-A receptor alpha 2 (ratio 4.75); GABA-A receptor beta 3 (ratio 2.25); GABA transporter 1 (ratio 2.30) in the striatum after exposure to DHCP, at 4 weeks<br>Upregulation of GABA-A receptor gamma 2 (ratio 1.60); GABA-B receptor 1a + 1b (ratio 1.80); GABA transporter 2 (ratio 1.70) in MID after exposure to DHCP, at 4 weeks<br>Upregulation of 5-HT receptor 4 (ratio 1.75); ionotropic Glu receptor 1 (ratio 4.60), ionotropic Glu receptor 3 (ratio 2.00); GABA-A receptor delta (ratio 1.62) in the striatum after exposure to DHCP, at 8 weeks<br>Upregulation of ionotropic Glu receptor 5 (ratio 2.00); metabotropic Glu receptor 4 (ratio 2.00); metabotropic Glu receptor 7 (ratio 2.30); GABA-A receptor alpha 1 (ratio 1.60) in the midbrain after exposure to DHCP, at 8 weeks<br>Upregulation of 5-HT receptor 1F (ratio 1.50); Glu transporters (ratio 1.30-1.70) in the midbrain after exposure to DCHP, at 4 weeks and 8 weeks<br>Downregulation of DA receptor D4 (ratio 0.22); metabotropic Glu receptor 5 (ratio 0.20) in the striatum after exposure to DHCP, at 4 weeks<br>Downregulation of DA transporter (ratio 0.50); ionotropic Glu receptor 1 (ratio 0.50); ionotropic Glu receptor epsilon 3 (ratio 0.40); GABA-A receptor gamma 1 (ratio 0.50); GABA transporter 1 (ratio 0.40) in MID after exposure to DHCP, at 4 weeks<br>Downregulation of GABA transporter 2 (ratio 0.46) in the midbrain after exposure to DHCP, at 8 weeks							

Table S3. *Cont.*

Source	Method							Outcome
	Population	N	Exposure			Analysis		
	Experimental group		Compound	Dose	Length	Location/ Technique	Time	
Masuo, Y. *et al*., 2004 [58]	Male Wistar rats	Not reported	DEHP	87 nmol/10 µL	Single dose (PND5)	In striatum and MID using macroarray	Freezing at 8 weeks of age, TOA not reported	Gene expression of: DA receptor drd1a, drd2, drd4, DA transporter DAT1, Glu receptor grin1, Glu transporter glast
	**Result:** Downregulation of drd1a (ratio 0.48) and grin1 (ratio 0.41) in MID after exposure to DEHP<br>Upregulation of glast in MID after exposure to DEHP (ratio 2.30)<br>Downregulation of grin1 in the striatum after exposure to DEHP (ratio 0.10)							
Masuo, Y. *et al*., 2004 [59]	Male Wistar rats	Not reported	DEHP, DBP	87 nmol/10 µL	Single dose (PND5)	In striatum and MID using macroarray	Freezing at 8 weeks of age, TOA not reported	Gene expression of: GABA transporter gat3, Glu receptor grin1, Glu transporter glast, DA receptor 1 dat1 and DA transporter drd4
	**Result:** Downregulation of grin1 (ratio 0.10), gat3 (ratio 0.38), drd4 (ratio 0.20) in the striatum after exposure to DEHP (ratio 0.50) or DBP<br>Upregulation of grin1 (ratio 1.58) and drd4 (ratio 1.83) in MID after exposure to DEHP<br>Upregulation of glast in the midbrain after exposure to DEHP (ratio 2.10) and DBP (ratio 2.30)							

***** Only significant results are reported. In case no significant results were found at all, the phrase "No significant results were found" was added to the result section.

References

1. Elnar, A.A.; Diesel, B.; Desor, F.; Feidt, C.; Bouayed, J.; Kiemer, A.K.; Soulimani, R. Neurodevelopmental and behavioral toxicity via lactational exposure to the sum of six indicator non-dioxin-like-polychlorinated biphenyls (summation operator6 NDL-PCBs) in mice. *Toxicology* **2012**, *299*, 44–54.
2. Dickerson, S.M.; Cunningham, S.L.; Gore, A.C. Prenatal pcbs disrupt early neuroendocrine development of the rat hypothalamus. *Toxicol. Appl. Pharmacol.* **2011**, *252*, 36–46.
3. Boix, J.; Cauli, O.; Felipo, V. Developmental exposure to polychlorinated biphenyls 52, 138 or 180 affects differentially learning or motor coordination in adult rats. Mechanisms involved. *Neuroscience* **2010**, *167*, 994–1003.
4. Martyniuk, C.J.; Feswick, A.; Spade, D.J.; Kroll, K.J.; Barber, D.S.; Denslow, N.D. Effects of acute dieldrin exposure on neurotransmitters and global gene transcription in largemouth bass (*Micropterus salmoides*) hypothalamus. *Neurotoxicology* **2010**, *31*, 356–366.
5. Cabaleiro, T.; Caride, A.; Romero, A.; Lafuente, A. Effects of *in utero* and lactational exposure to endosulfan in prefrontal cortex of male rats. *Toxicol. Lett.* **2008**, *176*, 58–67.
6. Babot, Z.; Vilaro, M.T.; Sunol, C. Long-term exposure to dieldrin reduces gamma-aminobutyric acid type a and n-methyl-d-aspartate receptor function in primary cultures of mouse cerebellar granule cells. *J. Neurosci. Res.* **2007**, *85*, 3687–3695.
7. Lafuente, A.; Cabaleiro, T.; Caride, A.; Gutierrez, A.; Esquifino, A.I. Toxic effects of methoxychlor in rat striatum: Modifications in several neurotransmitters. *J. Physiol. Biochem.* **2007**, *63*, 171–177.
8. Yuk, J.; Simpson, M.J.; Simpson, A.J. 1-D and 2-D NMR-based metabolomics of earthworms exposed to endosulfan and endosulfan sulfate in soil. *Environ. Pollut.* **2013**, *175*, 35–44.
9. Hilgier, W.; Lazarewicz, J.W.; Struzynska, L.; Frontczak-Baniewicz, M.; Albrecht, J. Repeated exposure of adult rats to Aroclor 1254 induces neuronal injury and impairs the neurochemical manifestations of the NMDA receptor-mediated intracellular signaling in the hippocampus. *Neurotoxicology* **2012**, *33*, 16–22.
10. Struzynska, L.; Sulkowski, G.; Dabrowska-Bouta, B. Aroclor 1254 selectively inhibits expression of glial GLT-1 glutamate transporter in the forebrain of chronically exposed adult rat. *Toxicology* **2012**, *300*, 12–18.
11. Boix, J.; Cauli, O.; Leslie, H.; Felipo, V. Differential long-term effects of developmental exposure to polychlorinated biphenyls 52, 138 or 180 on motor activity and neurotransmission. Gender dependence and mechanisms involved. *Neurochem. Int.* **2011**, *58*, 69–77.
12. Stavenes Andersen, I.; Voie, O.A.; Fonnum, F.; Mariussen, E. Effects of methyl mercury in combination with polychlorinated biphenyls and brominated flame retardants on the uptake of glutamate in rat brain synaptosomes: A mathematical approach for the study of mixtures. *Toxicol. Sci.* **2009**, *112*, 175–184.
13. Honma, T.; Suda, M.; Miyagawa, M.; Wang, R.S.; Kobayashi, K.; Sekiguchi, S. Alteration of brain neurotransmitters in female rat offspring induced by prenatal administration of 16 and 64 mg/kg of 2,2′,4,4′,5,5′-hexachlorobiphenyl (PCB153). *Ind. Health* **2009**, *47*, 11–21.

14. Lafuente, A.; Cabaleiro, T.; Caride, A.; Esquifino, A.I. Toxic effects of methoxychlor administered subcutaneously on the hypothalamic-pituitary-testicular axis in adult rats. *Food Chem. Toxicol.* **2008**, *46*, 1570–1575.
15. Castoldi, A.F.; Blandini, F.; Randine, G.; Samuele, A.; Manzo, L.; Coccini, T. Brain monoaminergic neurotransmission parameters in weanling rats after perinatal exposure to methylmercury and 2,2′,4,4′,5,5′-hexachlorobiphenyl (PCB153). *Brain Res.* **2006**, *1112*, 91–98.
16. Khan, I.A.; Thomas, P. Aroclor 1254 inhibits tryptophan hydroxylase activity in rat brain. *Arch. Toxicol.* **2004**, *78*, 316–320.
17. Lee, J.E.; Park, J.H.; Shin, I.C.; Koh, H.C. Reactive oxygen species regulated mitochondria-mediated apoptosis in pc12 cells exposed to chlorpyrifos. *Toxicol. Appl. Pharmacol.* **2012**, *263*, 148–162.
18. Coccini, T.; Roda, E.; Castoldi, A.F.; Poli, D.; Goldoni, M.; Vettori, M.V.; Mutti, A.; Manzo, L. Developmental exposure to methylmercury and 2,2′,4,4′,5,5′-hexachlorobiphenyl (PCB153) affects cerebral dopamine d1-like and d2-like receptors of weanling and pubertal rats. *Arch. Toxicol.* **2011**, *85*, 1281–1294.
19. Tian, Y.H.; Hwan, K.S.; Lee, S.Y.; Jang, C.G. Lactational and postnatal exposure to polychlorinated biphenyls induces sex-specific anxiolytic behavior and cognitive deficit in mice offspring. *Synapse* **2011**, *65*, 1032–1041.
20. Dreiem, A.; Okoniewski, R.J.; Brosch, K.O.; Miller, V.M.; Seegal, R.F. Polychlorinated biphenyls and polybrominated diphenyl ethers alter striatal dopamine neurochemistry in synaptosomes from developing rats in an additive manner. *Toxicol. Sci.* **2010**, *118*, 150–159.
21. Faro, L.R.; Alfonso, M.; Cervantes, R.; Duran, R. Comparative effects of pesticides on *in vivo* dopamine release in freely moving rats. *Basic Clin. Pharmacol. Toxicol.* **2009**, *105*, 395–400.
22. Schuh, R.A.; Richardson, J.R.; Gupta, R.K.; Flaws, J.A.; Fiskum, G. Effects of the organochlorine pesticide methoxychlor on dopamine metabolites and transporters in the mouse brain. *Neurotoxicology* **2009**, *30*, 274–280.
23. Gash, D.M.; Rutland, K.; Hudson, N.L.; Sullivan, P.G.; Bing, G.; Cass, W.A.; Pandya, J.D.; Liu, M.; Choi, D.Y.; Hunter, R.L.; *et al.* Trichloroethylene: Parkinsonism and complex 1 mitochondrial neurotoxicity. *Ann. Neurol.* **2008**, *63*, 184–192.
24. Hatcher, J.M.; Richardson, J.R.; Guillot, T.S.; McCormack, A.L.; Di Monte, D.A.; Jones, D.P.; Pennell, K.D.; Miller, G.W. Dieldrin exposure induces oxidative damage in the mouse nigrostriatal dopamine system. *Exp. Neurol.* **2007**, *204*, 619–630.
25. Lyng, G.D.; Snyder-Keller, A.; Seegal, R.F. Polychlorinated biphenyl-induced neurotoxicity in organotypic cocultures of developing rat ventral mesencephalon and striatum. *Toxicol. Sci.* **2007**, *97*, 128–139.
26. Caudle, W.M.; Richardson, J.R.; Delea, K.C.; Guillot, T.S.; Wang, M.; Pennell, K.D.; Miller, G.W. Polychlorinated biphenyl-induced reduction of dopamine transporter expression as a precursor to Parkinson's disease-associated dopamine toxicity. *Toxicol. Sci.* **2006**, *92*, 490–499.
27. Richardson, J.R.; Caudle, W.M.; Wang, M.; Dean, E.D.; Pennell, K.D.; Miller, G.W. Developmental exposure to the pesticide dieldrin alters the dopamine system and increases neurotoxicity in an animal model of Parkinson's disease. *FASEB J.* **2006**, *20*, 1695–1697.

28. Vettori, M.V.; Goldoni, M.; Caglieri, A.; Poli, D.; Folesani, G.; Ceccatelli, S.; Mutti, A. Antagonistic effects of methyl-mercury and pcb153 on pc12 cells after a combined and simultaneous exposure. *Food Chem. Toxicol.* **2006**, *44*, 1505–1512.
29. Jelaso, A.M.; DeLong, C.; Means, J.; Ide, C.F. Dietary exposure to aroclor 1254 alters gene expression in xenopus laevis frogs. *Environ. Res.* **2005**, *98*, 64–72.
30. Seegal, R.F.; Brosch, K.O.; Okoniewski, R.J. Coplanar PCB congeners increase uterine weight and frontal cortical dopamine in the developing rat: Implications for developmental neurotoxicity. *Toxicol. Sci.* **2005**, *86*, 125–131.
31. Bemis, J.C.; Seegal, R.F. PCB-induced inhibition of the vesicular monoamine transporter predicts reductions in synaptosomal dopamine content. *Toxicol. Sci.* **2004**, *80*, 288–295.
32. Lee, D.W.; Opanashuk, L.A. Polychlorinated biphenyl mixture aroclor 1254-induced oxidative stress plays a role in dopaminergic cell injury. *Neurotoxicology* **2004**, *25*, 925–939.
33. Richardson, J.R.; Miller, G.W. Acute exposure to aroclor 1016 or 1260 differentially affects dopamine transporter and vesicular monoamine transporter 2 levels. *Toxicol. Lett.* **2004**, *148*, 29–40.
34. Montes de Oca, L.; Moreno, M.; Cardona, D.; Campa, L.; Sunol, C.; Galofre, M.; Flores, P.; Sanchez-Santed, F. Long term compulsivity on the 5-choice serial reaction time task after acute chlorpyrifos exposure. *Toxicol. Lett.* **2013**, *216*, 73–85.
35. Noriega-Ortega, B.R.; Armienta-Aldana, E.; Cervantes-Pompa, J.A.; Hernandez-Ruiz, E.; Chaparro-Huerta, V.; Bravo-Cuellar, A.; Beas-Zarate, C. Gaba and dopamine release from different brain regions in mice with chronic exposure to organophosphate methamidophos. *J. Toxicol. Pathol.* **2011**, *24*, 163–168.
36. Pourabdolhossein, F.; Ghasemi, A.; Shahroukhi, A.; Sherafat, M.A.; Khoshbaten, A.; Asgari, A. *In vitro* assessment of paraoxon effects on gaba uptake in rat hippocampal synaptosomes. *Toxicol. In Vitro* **2009**, *23*, 868–873.
37. Mohammadi, M.; Ghani, E.; Ghasemi, A.; Khoshbaten, A.; Asgari, A. Synaptosomal GABA uptake decreases in paraoxon-treated rat brain. *Toxicology* **2008**, *244*, 42–48.
38. Shahroukhi, A.; Ghasemi, A.; Poorabdolhossein, F.; Asgari, A.; Khoshbaten, A. The effect of paraoxon on GABA uptake in rat cerebellar synaptosomes. *Med. Sci. Monit.* **2007**, *13*, BR194–BR199.
39. Ghasemi, A.; Sadidi, A.; Mohammadi, M.; Khoshbaten, A.; Asgari, A. Paraoxon inhibits GABA uptake in brain synaptosomes. *Toxicol. In Vitro* **2007**, *21*, 1499–1504.
40. Rush, T.; Liu, X.Q.; Hjelmhaug, J.; Lobner, D. Mechanisms of chlorpyrifos and diazinon induced neurotoxicity in cortical culture. *Neuroscience* **2010**, *166*, 899–906.
41. Xu, L.; Tian, H.; Wang, W.; Ru, S. Effects of monocrotophos pesticide on serotonin metabolism during early development in the sea urchin, hemicentrotus pulcherrimus. *Environ. Toxicol. Pharmacol.* **2012**, *34*, 537–547.
42. Eddins, D.; Cerutti, D.; Williams, P.; Linney, E.; Levin, E.D. Zebrafish provide a sensitive model of persisting neurobehavioral effects of developmental chlorpyrifos exposure: Comparison with nicotine and pilocarpine effects and relationship to dopamine deficits. *Neurotoxicol. Teratol.* **2010**, *32*, 99–108.

43. Moreno, M.; Canadas, F.; Cardona, D.; Sunol, C.; Campa, L.; Sanchez-Amate, M.C.; Flores, P.; Sanchez-Santed, F. Long-term monoamine changes in the striatum and nucleus accumbens after acute chlorpyrifos exposure. *Toxicol. Lett.* **2008**, *176*, 162–167.
44. Slotkin, T.A.; Ryde, I.T.; Levin, E.D.; Seidler, F.J. Developmental neurotoxicity of low dose diazinon exposure of neonatal rats: Effects on serotonin systems in adolescence and adulthood. *Brain Res. Bull.* **2008**, *75*, 640–647.
45. Slotkin, T.A.; Seidler, F.J. Prenatal chlorpyrifos exposure elicits presynaptic serotonergic and dopaminergic hyperactivity at adolescence: Critical periods for regional and sex-selective effects. *Reprod. Toxicol.* **2007**, *23*, 421–427.
46. Slotkin, T.A.; Seidler, F.J. Developmental exposure to terbutaline and chlorpyrifos, separately or sequentially, elicits presynaptic serotonergic hyperactivity in juvenile and adolescent rats. *Brain Res. Bull.* **2007**, *73*, 301–309.
47. Aldridge, J.E.; Meyer, A.; Seidler, F.J.; Slotkin, T.A. Alterations in central nervous system serotonergic and dopaminergic synaptic activity in adulthood after prenatal or neonatal chlorpyrifos exposure. *Environ. Health Perspect.* **2005**, *113*, 1027–1031.
48. Binukumar, B.K.; Gupta, N.; Bal, A.; Gill, K.D. Protection of dichlorvos induced oxidative stress and nigrostriatal neuronal death by chronic coenzyme q10 pretreatment. *Toxicol. Appl. Pharmacol.* **2011**, *256*, 73–82.
49. Chen, X.P.; Wang, X.; Dong, J.Y. Different reaction patterns of dopamine content to prenatal exposure to chlorpyrifos in different periods. *J. Appl. Toxicol.* **2011**, *31*, 355–359.
50. Sledge, D.; Yen, J.; Morton, T.; Dishaw, L.; Petro, A.; Donerly, S.; Linney, E.; Levin, E.D. Critical duration of exposure for developmental chlorpyrifos-induced neurobehavioral toxicity. *Neurotoxicol. Teratol.* **2011**, *33*, 742–751.
51. Binukumar, B.K.; Bal, A.; Kandimalla, R.J.; Gill, K.D. Nigrostriatal neuronal death following chronic dichlorvos exposure: Crosstalk between mitochondrial impairments, alpha synuclein aggregation, oxidative damage and behavioral changes. *Mol. Brain* **2010**, *3*, 35.
52. Eells, J.B.; Brown, T. Repeated developmental exposure to chlorpyrifos and methyl parathion causes persistent alterations in nicotinic acetylcholine subunit MRNA expression with chlorpyrifos altering dopamine metabolite levels. *Neurotoxicol. Teratol.* **2009**, *31*, 98–103.
53. Damodaran, T.V.; Patel, A.G.; Greenfield, S.T.; Dressman, H.K.; Lin, S.M.; Abou-Donia, M.B. Gene expression profiles of the rat brain both immediately and 3 months following acute sarin exposure. *Biochem. Pharmacol.* **2006**, *71*, 497–520.
54. Padilla, S.; Marshall, R.S.; Hunter, D.L.; Oxendine, S.; Moser, V.C.; Southerland, S.B.; Mailman, R.B. Neurochemical effects of chronic dietary and repeated high-level acute exposure to chlorpyrifos in rats. *Toxicol. Sci.* **2005**, *88*, 161–171.
55. Carbone, S.; Samaniego, Y.A.; Cutrera, R.; Reynoso, R.; Cardoso, N.; Scacchi, P.; Moguilevsky, J.A.; Ponzo, O.J. Different effects by sex on hypothalamic-pituitary axis of prepubertal offspring rats produced by *in utero* and lactational exposure to di-(2-ethylhexyl) phthalate (dehp). *Neurotoxicology* **2012**, *33*, 78–84.

56. Carbone, S.; Szwarcfarb, B.; Ponzo, O.; Reynoso, R.; Cardoso, N.; Deguiz, L.; Moguilevsky, J.A.; Scacchi, P. Impact of gestational and lactational phthalate exposure on hypothalamic content of amino acid neurotransmitters and FSH secretion in peripubertal male rats. *Neurotoxicology* **2010**, *31*, 747–751.
57. Ishido, M.; Masuo, Y.; Sayato-Suzuki, J.; Oka, S.; Niki, E.; Morita, M. Dicyclohexylphthalate causes hyperactivity in the rat concomitantly with impairment of tyrosine hydroxylase immunoreactivity. *J. Neurochem.* **2004**, *91*, 69–76.
58. Masuo, Y.; Ishido, M.; Morita, M.; Oka, S. Effects of neonatal treatment with 6-hydroxydopamine and endocrine disruptors on motor activity and gene expression in rats. *Neural. Plast.* **2004**, *11*, 59–76.
59. Masuo, Y.; Morita, M.; Oka, S.; Ishido, M. Motor hyperactivity caused by a deficit in dopaminergic neurons and the effects of endocrine disruptors: A study inspired by the physiological roles of PACAP in the brain. *Regul. Pept.* **2004**, *123*, 225–234.

© 2013 by the authors; licensee MDPI, Basel, Switzerland. This article is an open access article distributed under the terms and conditions of the Creative Commons Attribution license (http://creativecommons.org/licenses/by/3.0/).
